# Integrated computational and *Drosophila* cancer model platform captures previously unappreciated chemicals perturbing a kinase network

**DOI:** 10.1371/journal.pcbi.1006878

**Published:** 2019-04-26

**Authors:** Peter M. U. Ung, Masahiro Sonoshita, Alex P. Scopton, Arvin C. Dar, Ross L. Cagan, Avner Schlessinger

**Affiliations:** 1 Department of Pharmacological Sciences, Icahn School of Medicine at Mount Sinai, New York, NY, United States of America; 2 Department of Cell, Developmental and Regenerative Biology, Icahn School of Medicine at Mount Sinai, New York, NY, United States of America; 3 Department of Oncological Sciences, Icahn School of Medicine at Mount Sinai, New York, NY, United States of America; Johns Hopkins University, UNITED STATES

## Abstract

*Drosophila* provides an inexpensive and quantitative platform for measuring whole animal drug response. A complementary approach is virtual screening, where chemical libraries can be efficiently screened against protein target(s). Here, we present a unique discovery platform integrating structure-based modeling with *Drosophila* biology and organic synthesis. We demonstrate this platform by developing chemicals targeting a *Drosophila* model of Medullary Thyroid Cancer (MTC) characterized by a transformation network activated by oncogenic dRet^M955T^. Structural models for kinases relevant to MTC were generated for virtual screening to identify unique preliminary hits that suppressed dRet^M955T^-induced transformation. We then combined features from our hits with those of known inhibitors to create a ‘hybrid’ molecule with improved suppression of dRet^M955T^ transformation. Our platform provides a framework to efficiently explore novel kinase inhibitors outside of explored inhibitor chemical space that are effective in inhibiting cancer networks while minimizing whole body toxicity.

## Author summary

Effective and safe treatment of multigenic diseases often involves drugs that address multiple points along disease networks, i.e., polypharmacology. Polypharmacology is increasingly appreciated as a potentially desirable property of kinase drugs. However, most known drugs that interact with multiple targets have been identified as such by chance and most polypharmacological compounds are not chemically unique, resembling structures of known kinase inhibitors. The fruit fly *Drosophila* provides an inexpensive, rapid, quantitative, whole animal screening platform that has the potential to complement computational approaches. We present a chemical genetics approach that efficiently combines *Drosophila* with structural prediction and virtual screening, creating a unique discovery platform. We demonstrate the utility of our approach by developing useful small molecules targeting a kinase network in a *Drosophila* model of Medullary Thyroid Cancer (MTC) driven by oncogenic dRet^M955T^.

## Introduction

Protein kinases play a key role in cell signaling and disease networks and represent major therapeutic targets. The limited capacity to test large numbers of compounds to explore diverse chemical scaffolds, coupled with the difficulty in translating *in vitro* kinase inhibition into whole animal efficacy, has limited the chemical space of the known kinase inhibitors (KIs). As a result, obtaining optimal KIs with clinically relevant therapeutic activity has proven challenging despite extensive academic and industry effort.

To expand the number of kinase inhibitors, a variety of platforms have recently emerged as useful tools for compound screening. The fruit fly *Drosophila melanogaster* provides an inexpensive and efficient whole animal platform for cancer drug screening, capturing clinically relevant compounds [1–3]. For example, *Drosophila* was used to help validate vandetanib as a useful treatment for medullary thyroid cancer [4] (MTC). As a screening platform, *Drosophila* offers several advantages: First, flies and humans share similar kinome and kinase-driven signaling pathways [5], facilitating the use of flies to predict drug response in humans [1, 6]. Second, the ease of breeding and the short (~10 day) life cycle of *Drosophila* makes it possible to carry out efficient moderate-throughput chemical screening in a whole animal system. Third, the screening readout provides a quantitative, animal-based measurement of structure-activity relationships (SAR) as well as information on the therapeutic potential or toxicity of the tested compounds: measurable parameters include survival and multiple phenotypic indicators that depend on kinase activity.

A key limitation of a *Drosophila*-based moderate-throughput screening platform is its inability to explore very large chemical libraries [7] such as the ZINC library, which has over 750 million purchasable compounds [8]. In contrast, structure-based virtual screening is a fast and inexpensive computational method that can screen large compound libraries, useful for identifying unique chemical probes [9]. If the structure of the protein is unknown, virtual screening can be performed against the homology models of the target based on experimentally determined structures. However, the automated construction of homology models—with sufficient accuracy for simultaneous virtual screening of multiple targets and the application of molecular docking to signaling networks—remains challenging in particular for highly dynamic targets such as kinases [10, 11] and would benefit from a readily accessible whole animal platform. In this paper, we demonstrate how combining *Drosophila* and computational approaches provides a synergistic platform for lead compound discovery, combining the strengths of computational methods—which enable rational and rapid drug candidate selection—and a *Drosophila* animal model that enables fast and relevant biological readouts of tested compounds. We demonstrate the practicality of this approach using MTC as a test case.

RET is a receptor tyrosine kinase associated with multiple roles in development and disease. The gain-of-function M918T mutation of RET (analogous to *Drosophila* M955T) activates multiple proliferation pathways and is directly associated with MTC pathogenesis [12, 13]. Transgenic *Drosophila* expressing the analogous dRet^M955T^ isoform show key aspects of transformation, including proliferation and aspects of metastasis [6, 14]. Genetic modifier screens with dRet^M955T^ flies led to the identification of multiple RET pathway genetic ‘suppressors’ and ‘enhancers’, loci that when reduced in activity improve or worsen the dRet^M955T^ phenotype, respectively. These functional mediators of RET-dependent transformation include members of the Ras/ERK and PI3K pathways as well as regulators of metastasis such as SRC [6, 15].

**Fig 1:**
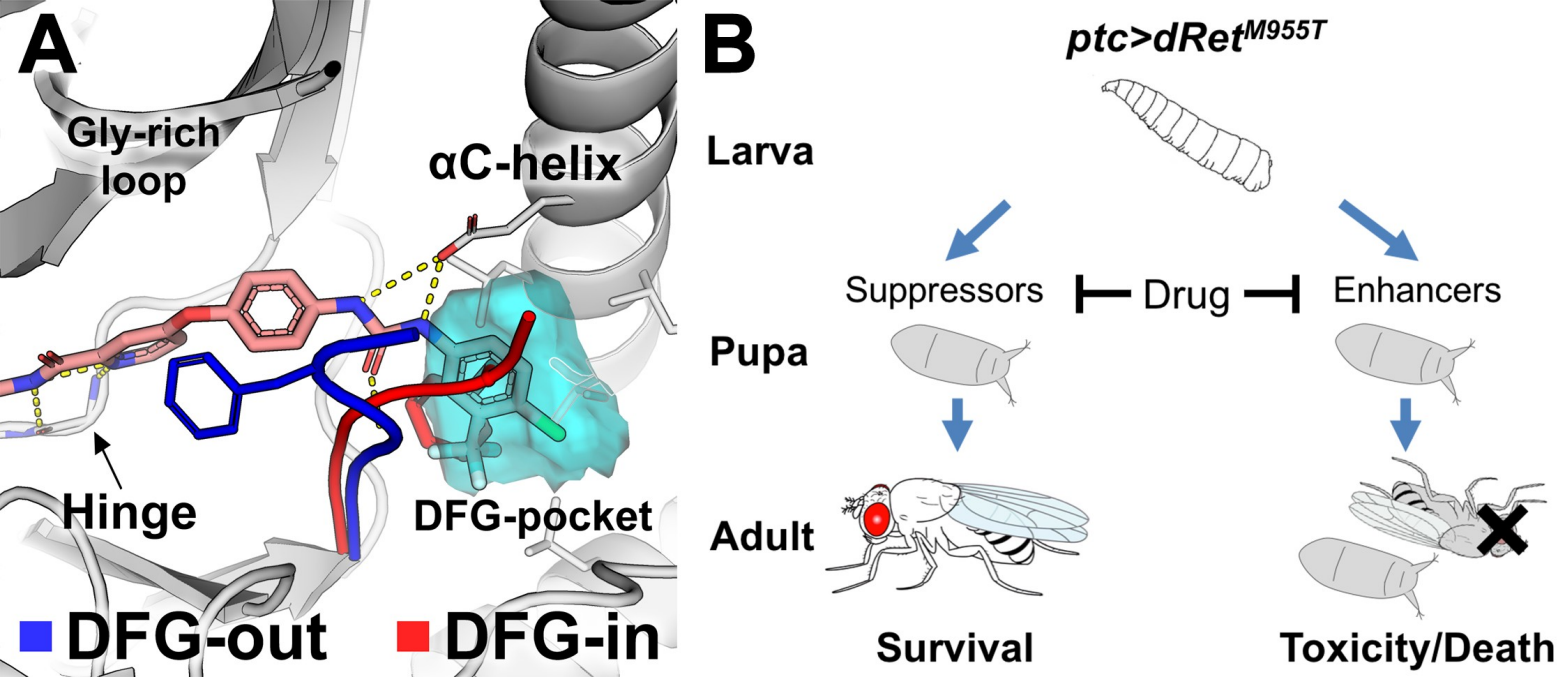
Kinase binding to type-II kinase inhibitors. (A) The conformational state of protein kinases (e.g., KDR) including DFG-in (red) and DFG-out (blue) is determined by the DFG-motif. The DFG-pocket (cyan mesh) is unique to the DFG-out conformation. Sorafenib is shown in pink. Broken yellow lines indicate hydrogen bonds. (B) A scheme depicting the positive and negative effects of drug acting on genetic modifiers of medullary thyroid cancer in a Drosophila model. ptc-driven dRet^M955T^ induces lethality during development. ‘Suppressors’ or ‘enhancers’ suppress or enhance, respectively, dRet^M955T^-induced disease phenotypes as revealed in genetic screening. A drug can suppress lethality by inhibiting the suppressors. It can also induce toxicity and/or worsen transformed phenotypes by inhibiting the enhancers, which results in enhanced lethality.

Oral administration of the FDA-approved multi-kinase inhibitor analogs sorafenib and regorafenib—along with additional structural analogs—partially rescued *dRet*^*M955T*^-induced transformation in *Drosophila* [1, 15]. Sorafenib-class inhibitors are classified as ‘type-II’ KIs that bind the kinase domain in their inactive state [16], a conformational state regulated by the aspartate-phenylalanine-glycine (DFG)-motif (Fig 1A) [17, 18]. In the inactive, ‘DFG-out’ conformational state the directions of DFG-Asp and DFG-Phe ‘flip’, vacating the DFG-Phe pocket (‘DFG-pocket’) that modulates binding to type-II inhibitors. A key challenge of targeting kinases in the DFG-out conformation with structure-based virtual screening is that few kinase structures have been reported with the DFG-out conformation [19, 20]. We recently developed DFGmodel [10], a computational method for modeling kinases in DFG-out conformations. This method informed the mechanism of clinically relevant multi-kinase inhibitors that target the MTC network [15].

**Fig 2:**
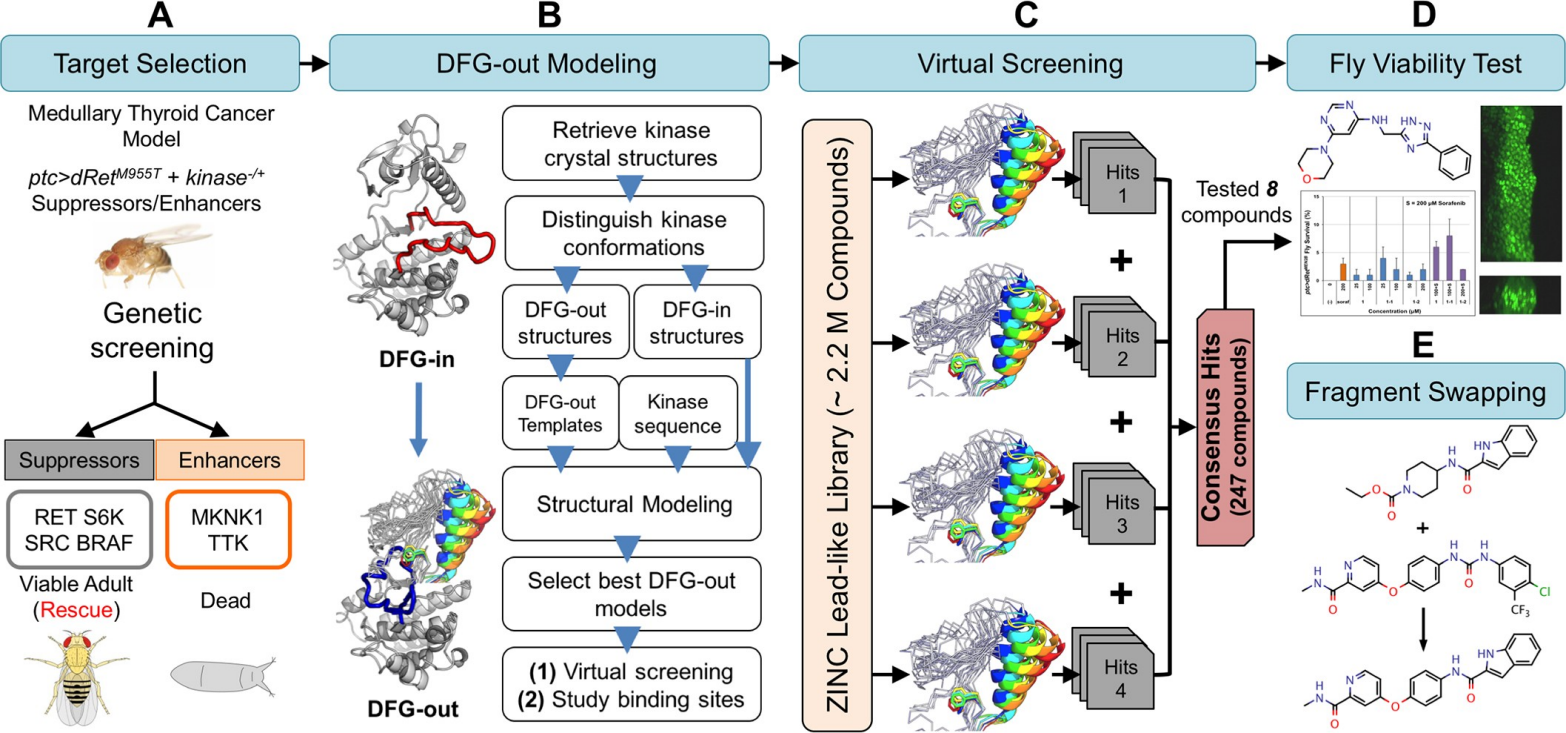
Fly genetics and computational chemistry discovery platform. Key steps include (A) determining suppressors and enhancers in a dominant modifier genetic screening and their in silico modelability, (B) generating DFG-out kinase models using DFGmodel, (C) virtual screenings of compound libraries against the modeled suppressors and enhancers, combining top-ranking screening results into consensus result, (D) testing top-ranking compounds for rescue of lethality (left panel) and migration of transformed cells in developing wing discs of *ptc>dRet^M955T^* flies (right panel), and (E) refining hits by combining structural elements of computationally derived hits and that of drugs and evaluating new targets.

In this study, we report the development of an integrated platform (Fig 2) that combines (i) computational modeling of kinases in their inactive state plus massive multi-target virtual screening with (ii) whole animal *Drosophila* assays to identify previously unappreciated chemicals that perturb RET-dependent transformation. This integrative platform combines the strengths of computational methods—including facilitating rational and rapid compound prioritization for experimental testing—and *Drosophila* models that provide a whole animal readout of compound efficacy. We leverage this integrated fly/computational modeling platform to create a novel ‘hybrid’ molecule with unique chemical structure and biological efficacy. Finally, we discuss the relevance of this approach to expedite the discovery of novel chemical scaffolds targeting disease networks.

## Results

### Target selection from fly genetic screen and structural analysis

In transgenic *patched-GAL4;UAS-dRet*^*M955T*^ (*ptc>dRet*^*M955T*^) flies, the *ptc* promoter drives expression of an oncogenic isoform of *Drosophila* Ret in multiple tissues; the result is lethality prior to adult eclosion [1, 15]. We previously used this and similar fly MTC models in genetic screens to identify ~40 kinases that mediate *dRet*^*M955T*^–mediated transformation [6, 15] (Figs 1B and 2A; S1 Fig).

To narrow this list, we prioritized candidate kinases based on two considerations: (i) pharmacological relevance as known mediators of RET signaling [6, 21]; (ii) structural coverage, specifically kinases with known DFG-out structures or those that can be modeled with sufficient accuracy [10]. Atypical kinases (*e*.*g*., mTOR and eEF2K) and members of the RGC family were excluded as they have diverse sequence and structure features that limit our ability to generate accurate homology models. Applying these criteria to our genetic modifier list, we focused on targeting four key kinase targets: RET (receptor tyrosine kinase), SRC (cytoplasmic tyrosine kinase), BRAF (tyrosine kinase-like), and p70-S6K (AGC family).

### Modeling kinases in DFG-out conformation

Description of the various conformations adopted by the kinases during activation and inhibition is needed for rationally designing novel, conformation-specific inhibitors. Therefore, our approach was to perform massive structure-based virtual screens of purchasable compound libraries against multiple models with DFG-out conformation; our goal was to identify generic kinase inhibitors that may target one or more prioritized kinases but, importantly, demonstrate an effect on the disease pathway in the animal model.

The structure of two of the kinases identified in our *dRet*^*M955T*^ genetic screens—BRAF and SRC—have been solved in the DFG-out conformation; the DFG-out structures of RET and p70-S6K have not been reported. We therefore generated DFG-out models using DFGmodel, a computational tool that generates homology models of kinase in DFG-out conformation through multiple-template modeling that samples a range of relevant conformations [10]. In our previous study, we tested and confirmed subsets of DFG-out models that enrich for known type-II inhibitors among a diverse set of non-type-II KIs found in the Protein Data Bank (PDB) with accuracy similar to or better than that obtained for experimentally determined structures [10]. For example, in a recent application of DFGmodel, models generated by this method were used in parallel with medicinal chemistry to optimize clinically relevant compounds that are based on the established kinase inhibitor sorafenib [15]. Conversely, in this study models generated by DFGmodel were used to develop compounds outside of the current kinase inhibitor chemical space.

**Fig 3:**
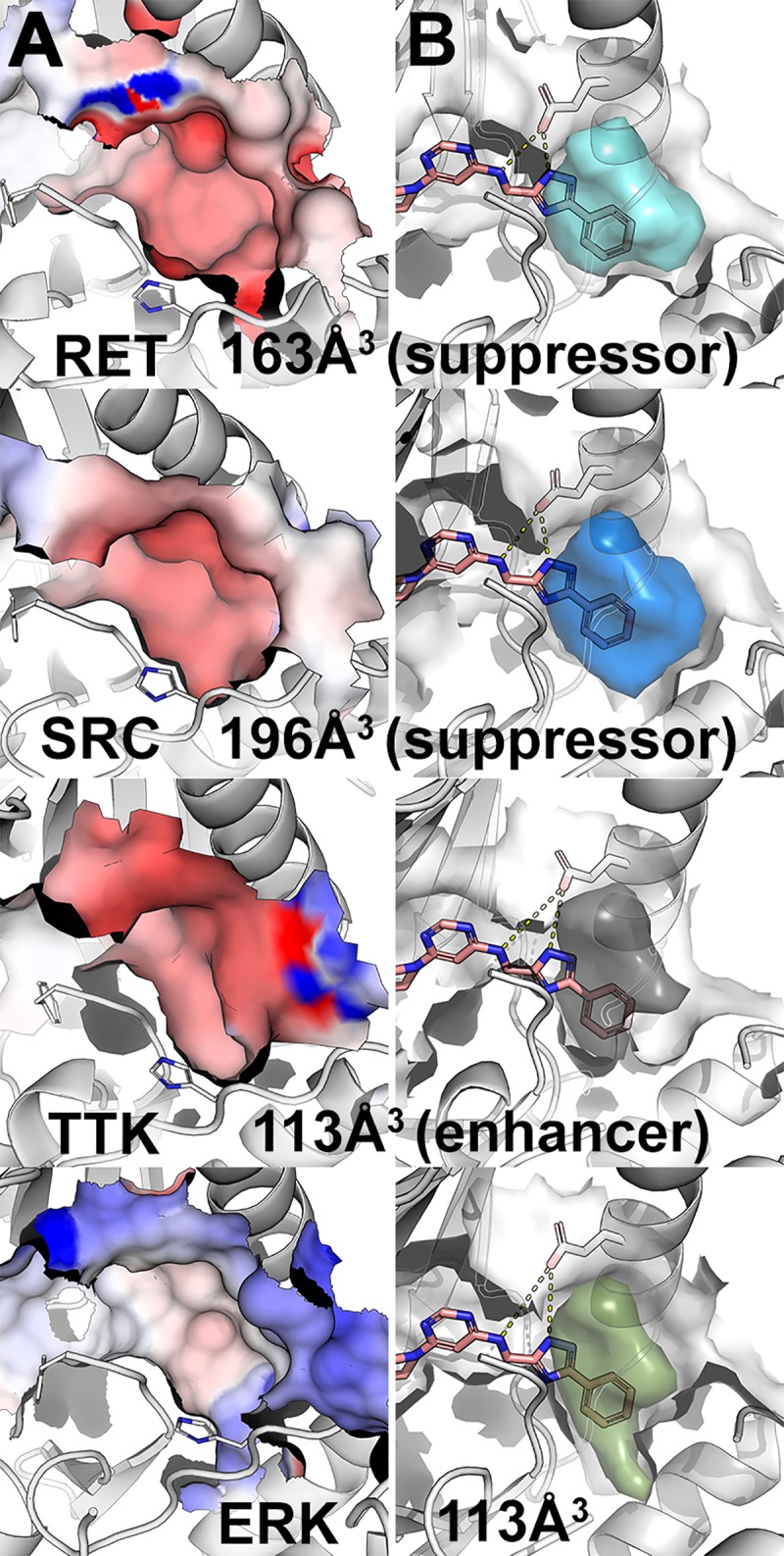
Visualization of DFG-pockets. (A) Electrostatic potential (red, negative potential; blue, positive potential) on the surface of the DFG-pocket in various kinases, including the suppressors RET and SRC, the enhancer TTK, and ERK. (B) Accessible volume of the DFG-pocket (colored volume) for potential type-II kinase inhibitor. Hit molecule 1 is depicted in pink sticks. Broken yellow lines indicate hydrogen bonds.

To guide the identification of a ‘generic’ kinase inhibitor that can affect a disease pathway we first compared the DFG-out models of each kinase, identifying key similarities and differences in physicochemical properties among their inhibitor-binding sites. First, we noted that the prioritized targets RET, BRAF, p70-S6K, and SRC present negative electrostatic potential on the DFG-pocket surface, while many non-targets such as ERK have positive electrostatic potential (Fig 3A, S2 Fig). This difference may partially explain the partial selectivity of type-II inhibitors (*e*.*g*., sorafenib) toward our prioritized targets while avoiding electrostatic positive kinases such as ERK. Second, RET and SRC have large DFG-pocket volumes (163 Å^3^, 196 Å^3^, respectively) and p70-S6K and BRAF have moderately large pockets (158 Å^3^, 136 Å^3^). In contrast, ERK has a small DFG-pocket (113 Å^3^; Fig 3B). We used this size difference to computationally select for kinases with larger DFG-pockets (e.g., RET, SRC) while excluding kinases with smaller DFG-pockets (*e*.*g*., ERK).

### Virtual screening against MTC pathway

We performed virtual screening against multiple DFG-out models of MTC targets to identify putative small molecules that modulate the disease network (Fig 2C). We docked a purchasable lead-like library from the ZINC database (2.2 millions compounds; [22]) against 10 DFG-out models for each kinase target, yielding over 88 million total docking poses. To combine the screening results, a two-step consensus approach was used. In the first step, the top scoring pose of compounds that ranked in the top 10% in 5 or more of the 10 models of each kinase were selected, resulting in approximately 2,000 compounds per kinase. In the second step, compounds that ranked in the top 25% in at least 3 of 4 targets were selected, resulting in 247 compounds. For comparison, sorafenib, an inhibitor that rescues *ptc>dRet*^*M955T*^ flies, would rank eighth in this consensus docking result. From these consensus compounds, eight commercially available compounds were purchased to test their ability to rescue *ptc>dRet*^*M955T*^ flies (S1 Table). These compounds were selected based on their interactions with key elements of the “ensemble” of targets’ binding sites, with the emphasis on the conserved glutamate in αC-helix, the amide backbone of DFG-aspartate, and if present, the amide backbone of the hinge region (S3 Fig). Although the compounds are not predicted to bind optimally to each one of our targets, we hypothesized that these compounds may have an additive effect on the disease pathway, which could be improved with medicinal chemistry.

### Testing candidates in *ptc>dRet^M955T^* fly viability assay

Transgenic *ptc>dRet*^*M955T*^ flies express the oncogenic *Drosophila* dRet^M955T^ isoform in several tissues in the developing fly, leading to aspects of transformation of dRet^M955T^ tissues [6, 14]. As a result, *ptc>dRet*^*M955T*^ flies exhibited 0% adult viability when cultured at 25°C, providing a quantitative ‘rescue-from-lethality’ assay to test drug efficacy [1, 15]. Compounds were fed at the highest accessible concentrations (see Experimental Procedures). We used sorafenib as a positive control, as it previously demonstrated the highest level of rescue among FDA-approved KIs in *ptc>dRet*^*M955T*^ flies [15]. Similar to our previous results, feeding *ptc>dRet*^*M955T*^ larvae sorafenib (200 μM) improved overall viability to 3–4% adult survival (*P* < 0.05).

**Fig 4:**
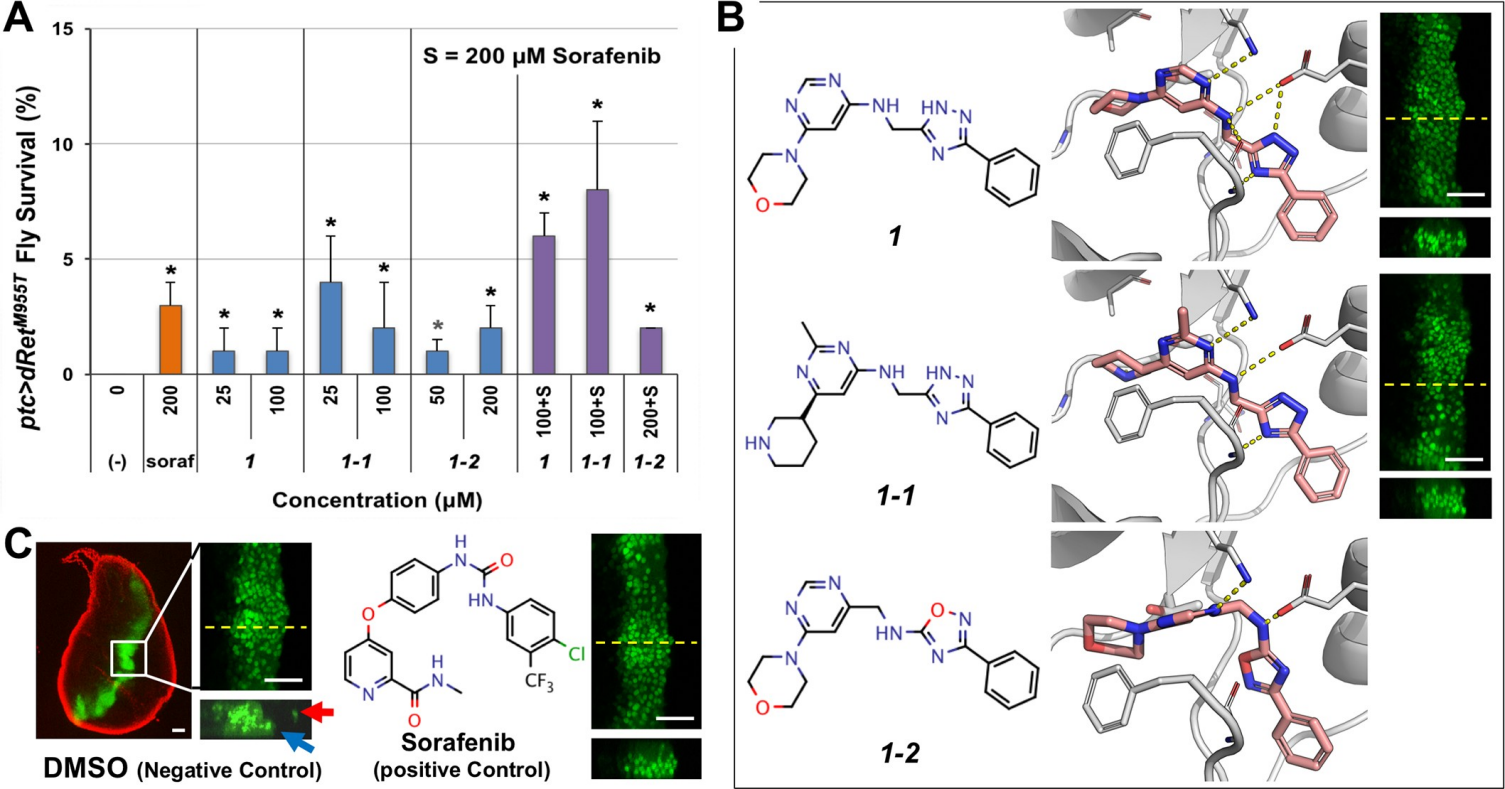
Compound 1 and its analogs. (A) Rescue of *ptc>dRet^M955T^* fly lethality by 1 and 1–1. Both showed improved efficacy (synergy) when co-administrated with 200 μM sorafenib (soraf). (-), vehicle DMSO control. Error bars represent standard error in triplicate experiments. *P < 0.05 in one-sided Student’s t-test as compared with vehicle control. (B) Docking pose of 1 and its analogs 1–1 and 1–2 (salmon sticks) with a DFG-out model of RET (broken yellow lines indicate hydrogen bonds), and their inhibition of migration of the dRet^M955T^-expressing cells. Right, suppression of cell migration by 1 and 1–1. Controls are shown in (C). (C) In vivo cell migration assay in *ptc>dRet^M955T^* flies. Left, a developing whole wing disc containing GFP-labeled, dRet^M955T^-expressing cells constituting a stripe in the midline. The disc margin is visualized with DAPI (red pseudocolor). There are wild-type cells in black areas. Center, overgrowth of dRet^M955T^-expressing cells resulting in the thickening of the stripe in the apical view (top). Virtual z-series view of confocal images derived from the plane indicated by yellow dotted lines (bottom) shows dRet^M955T^-expressing cells migrating away from the original domain (arrows). Right, sorafenib suppressed the migration. White scale bars, 50 μm.

**Fig 5:**
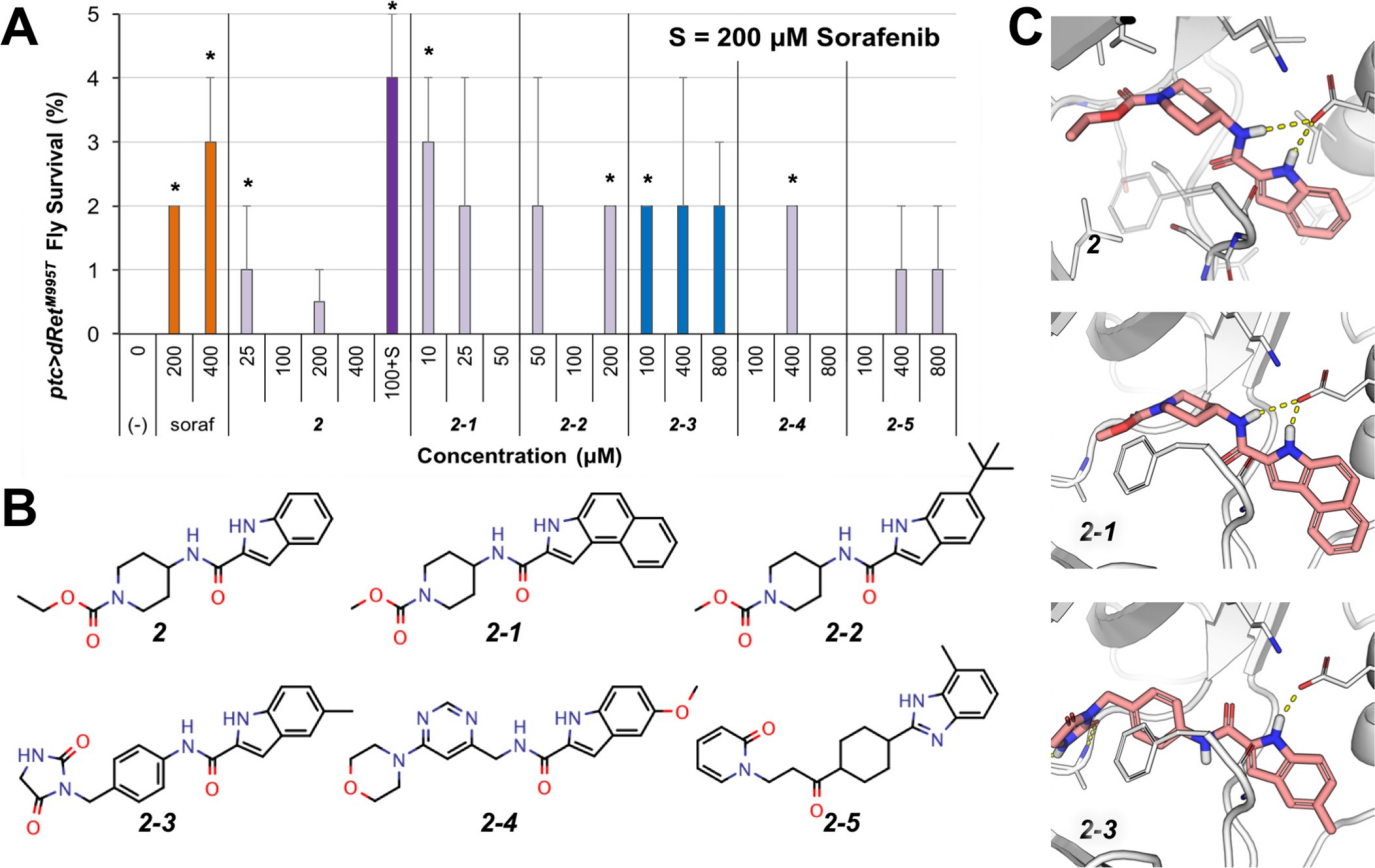
Rescue of *ptc>dRet^M955T^* flies by 2 and its analogs. (A) *ptc>dRet^M955T^* viability assay. 2 showed increased efficacy when co-administrated with 200 μM sorafenib. (-), vehicle control. Error bars represent standard error in triplicate experiments. *P < 0.05 in one-sided Student’s t-test as compared with no-drug control. (B) Chemical structure of 2 and its analogs. (C) Docking pose of 2 and its analogs in a RET DFG-out model. These compounds are proposed to be putative type-II kinase inhibitors that bind in the DFG-pocket through their 1H-indole moiety and interact with the conserved αC-helix glutamate side chain and DFG-Aspartate backbone (broken yellow lines).

We used this rescue-from-lethality assay to test the efficacy of the eight compounds identified through virtual screening (Figs 4B and 5B). When fed orally, two unique compounds, ***1*** and ***2*** (S2 Table), rescued a small fraction of *ptc>dRet*^*M955T*^ flies to adulthood (Figs 4A and 5A). ***1*** and ***2*** did not affect the body size of *ptc>dRet*^*M955T*^ larvae or pupae compared to wild type controls, a metric for comparing food intake. At the maximum final concentration in fly food (100 μM), ***1*** rescued 1% (*P* < 0.05) *ptc>dRet*^*M955T*^ flies to adulthood as compared to 3–4% rescue by sorafenib at 200 μM (Fig 4A). ***1*** is characterized by a 3-phenyl-(1*H*)-1,2,4-trazole moiety (Fig 4B). ***2***, characterized by a 1*H*-indole-2-carboxamide moiety, improved *ptc>dRet*^*M955T*^ fly viability to an average of 1% (*P* < 0.05) when tested at 25–400 μM (Fig 5A and 5B).

### Confirmation of novel chemical scaffolds

To validate the chemical scaffolds identified in our initial *Drosophila*-based chemical genetic screening, we conducted a ligand-based chemical similarity search in the updated ZINC [8] to identify analogs of ***1*** and ***2***. For compound ***1***, we retrieved five compounds that share the 3-phenyl-(1*H*)-1,2,4-triazole feature and have docking poses similar to ***1***. Our *Drosophila ptc>dRet*^*M955T*^ viability assay confirmed two compounds as active, ***1–1*** and ***1–2*** (S2 Table; Fig 4A and 4B). ***1–1*** slightly outperformed ***1*** in *ptc>dRet*^*M955T*^ viability assays at similar concentrations (4%; *P* < 0.05). Conversely, ***1–2*** was tested at higher concentrations (50 and 200 μM) but did not result in improved efficacy (*P* < 0.05).

The docking poses of ***1–1*** and ***1–2*** resemble the proposed docking pose of ***1*** (Fig 4B), which has a typical DFG-out-specific, type-II KI binding pose and is predicted to occupy the DFG-pocket with its terminal phenyl moiety. The 1,2,4-triazole moiety, resembles the urea moiety found in sorafenib (Fig 1A), forms favorable hydrogen bonds with the side chain of the conserved αC-helix glutamate residue and the backbone amide of the DFG-Aspartate. In addition, this series of compounds is smaller and shorter (MW < 360) than the fully developed type-II KIs (MW > 450) such as sorafenib, as they lack an optimized moiety that interacts with the hinge region of the ligand-binding site (Fig 4C).

Compound ***1–2*** is structurally different from ***1*** and ***1–1*** and was less effective in rescuing *ptc>dRet*^*M955T*^ flies, even though it was tested at higher concentrations (Fig 4A). While ***1*** and ***1–1*** have an (1*H*)-1,2,4-triazole moiety, ***1–2*** has an 1,2,4-oxadiazol-5-amine moiety where the (1*H*)-nitrogen is replaced by an oxygen. This modification distinguishes ***1–2*** from ***1*** and ***1–1*** in their interaction preference: ***1–2*** loses a hydrogen bond donor due to the nitrogen-to-oxygen substitution, while the electronegative oxygen introduces an unfavorable electrostatic repulsion to the carboxylate sidechain of the conserved αC-helix glutamate (Fig 4C, ***1–2*** insert).

Co-administering sorafenib with ***1*** and ***1–1*** led to synergistic improvement of *ptc>dRet*^*M955T*^ fly viability (Fig 4A). Individually, 200 μM of sorafenib and 100 μM of ***1*** rescued 3% and 1% of *ptc>dRet*^*M955T*^ flies to adulthood, respectively. Co-administering the two compounds rescued 6% of *ptc>dRet*^*M955T*^ flies to adulthood (*P* < 0.05). Similarly, co-administering sorafenib and 100 μM of ***1–1*** rescued 8% of *ptc>dRet*^*M955T*^ flies (*P* < 0.05). In contrast, co-administering 200 μM of sorafenib and 200 μM of ***1–2*** did not improve fly viability. As ***1–2*** only weakly rescued *ptc>dRet*^*M955T*^ flies and showed no synergy with sorafenib, we did not pursue this hit any further.

We examined the kinase inhibition profile (DiscoverX) of ***1*** against a subset of the human protein kinome (Table 1). At 50 μM, ***1*** did not appreciably inhibit SRC, BRAF, or S6K1, while it demonstrated weak activity against wild-type RET and moderate activity against the oncogenic isoform RET^M918T^. Of note, ***1*** inhibited other cancer-related targets such as FLT3 (Table 1), which activates the Ras/ERK signaling pathway [23].

**Table 1:** Kinase inhibition profile of compound 1 at 50 μM.

Kinase	% Inhib.	Kinase	% Inhib.
ABL1	0	mTOR	4
AURKA	22	PDGFRB	21
AURKB	7	RET	15
AURKC	2	RET (M918T)	28
BRAF	9	RET (V804L)	35
CSF1R	3	S6K1	0
FGFR	0	SRC	5
**FLT3**	**52**	TTK	17

Bold, inhibited by more than 40%.

***1*** also showed activity against aspects of transformation and metastasis in the fly. In the mature larva, the *ptc* promoter is active in epithelial cells in a stripe pattern in the midline of the developing wing epithelium (Fig 4C; wing disc). *ptc*-driven dRet activates multiple signaling pathways, promoting proliferation, epithelial-to-mesenchymal transition (EMT), and invasion of dRet^M955T^-expressing cells beyond the *ptc* domain [14] (Fig 4C). Similar to sorafenib, oral administration of ***1*** blocked the invasion of dRet^M955T^-expressing cells into the surrounding wing epithelium (Fig 4B).

At lower dosage (25 μM), compound ***2*** weakly rescued *ptc>dRet*^*M955T*^ flies (1%; *P* < 0.05) (Fig 5A). Unlike ***1***, ***2*** did not act synergistically with sorafenib. This difference was confirmed by the kinase inhibition profile of ***2*** (Table 2), in which it has stronger inhibition of RET and RET^M918T^, but loses the inhibition of FLT3, two key differences between the kinase inhibition profiles of *1* and *2*.

**Table 2:** Kinase inhibition profile of compounds 2 and 2–3 at 50 μM.

Compound *2*				Compound *2–3*			
Kinase	% Inhib.	Kinase	% Inhib.	Kinase	% Inhib.	Kinase	% Inhib.
ABL1	0	mTOR	5	ABL1	2	mTOR	8
AURKA	10	PDGFRB	20	AURKA	13	PDGFRB	**91**
AURKB	2	RET	34	AURKB	22	RET	24
AURKC	22	**RET (M918T)**	**44**	AURKC	2	RET (M918T)	23
BRAF	0	**RET (V804L)**	**44**	BRAF	0	**RET (V804L)**	**43**
CSF1R	12	S6K1	0	CSF1R	20	S6K1	0
FGFR	0	SRC	6	FGFR	4	SRC	0
FLT3	25	TTK	31	**FLT3**	**78**	TTK	26

Bold, inhibited by more than 40%.

Through a chemical similarity search of the ZINC database, we identified five compounds that share the 1*H*-indole-2-carboxamide moiety with docking poses similar to that of ***2*** (Fig 5B and 5C; S2 Table), and confirmed all five analogs increased viability of *ptc>dRet*^*M955T*^ flies (Fig 5A) albeit with weak efficacy (some with a *P*-value above 0.05). At low dose (10 μM), ***2–1*** showed improved efficacy in rescuing *ptc>dRet*^*M955T*^ flies relative to ***2*** and displayed similar efficacy as sorafenib at 200 μM. However, ***2–1*** showed poor solubility, limiting its usefulness as a lead compound. ***2–3*** was also more efficacious than ***2*** and displayed better solubility in both DMSO and water than ***2–1***; it also has the *N*-phenylacetamide moiety as a linker group, a common linker feature found in type-II KIs such as imatinib. Compound ***2–3*** displayed a different inhibition profile than ***1*** and ***2*** (Table 2): it strongly inhibits FLT3 and PDGFRB, weakly inhibits RET and RET^M918T^, and does not inhibit SRC.

### Improving efficacy through compound hybridization

Interestingly, the chemical scaffolds of our newly identified active compounds are not associated with inhibition of protein kinases based on an analysis with SEA search [24], which relates ligand chemical similarity of ligands to protein pharmacology. Nevertheless, they provided rescue of *ptc>dRet*^*M955T*^ flies at a level similar to sorafenib and regorafenib [15]. The docking poses of these compounds suggest a less-than-optimal interaction with the hinge region of protein kinases, a common feature of most KIs. Furthermore, the relatively low molecular weight (~350 g/mol) of these lead-like compounds provides a window for conducting lead optimization with medicinal chemistry. Hence, we sought to improve the efficacy of our computationally derived leads by installing a hinge-binding moiety found in known type-II KIs such as sorafenib.

**Fig 6:**
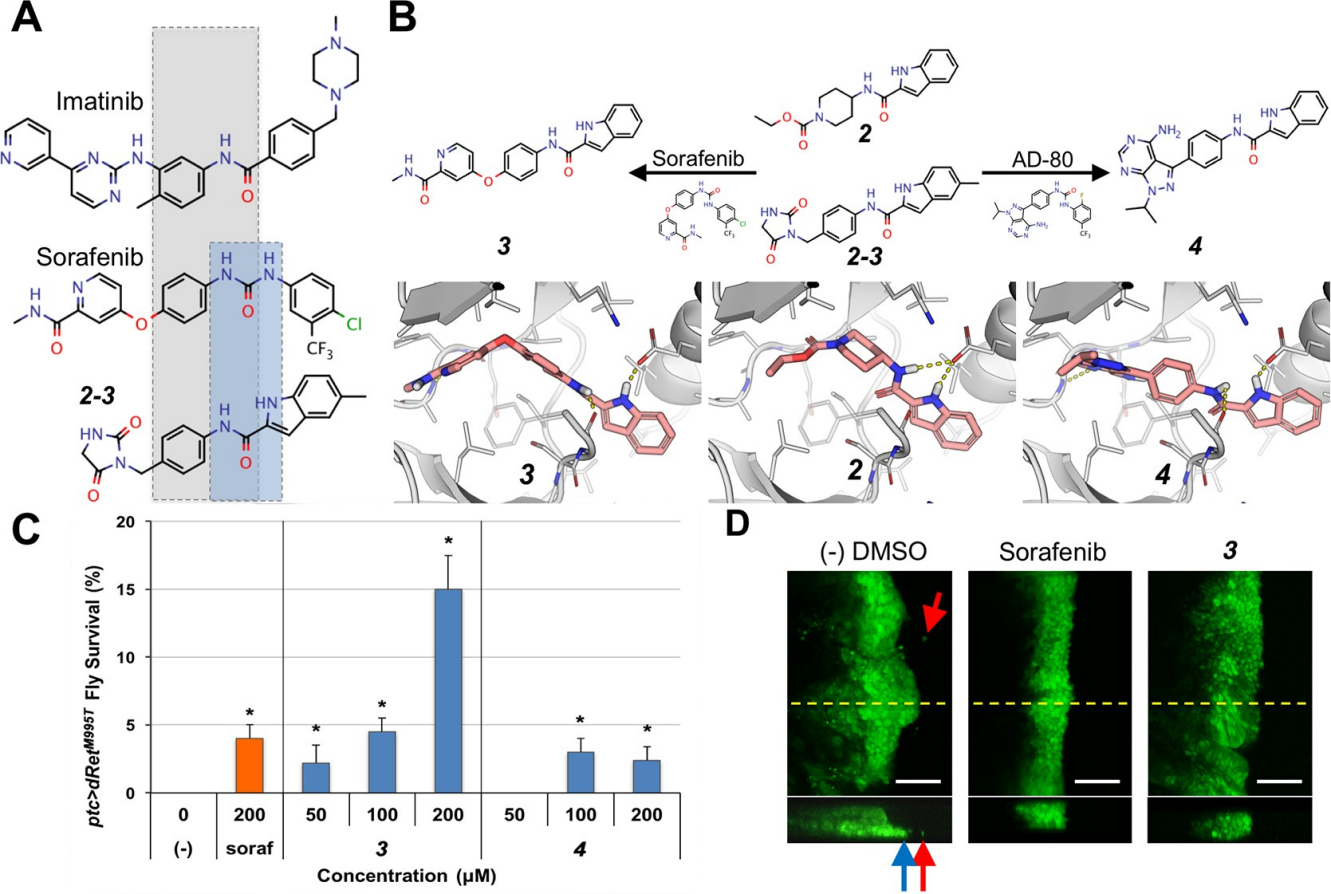
Hybrid compounds with improved efficacy. (A) The kinase inhibitors imatinib, sorafenib, and 2–3 share the common N-phenylcarboxamide moiety (grey box), while the 1H-indole-2-carboxamide of 2–3 resembles the urea linker of sorafenib (blue box). (B) Hybridization of 2 and sorafenib and AD-80 yielded 3 and 4, respectively. Top, chemical structures of compounds. Bottom, docking poses of compounds in a RET DFG-out model. (C) 3 rescued *ptc>dRet^M955T^* flies more effectively than by either 2 or sorafenib alone. (-), vehicle control. Error bars represent standard error in triplicate experiments. *P < 0.05 in one-sided Student’s t-test as compared with no-drug control. (D) 3 suppresses migration of dRet^M955T^-expressing wing disc cells from the original domain (green) similarly to the positive control, sorafenib. Top and bottom, apical and z-series views at the yellow dotted lines in apical view, respectively. Arrows, migrating cells. White scale bars, 50 μm.

To select the optimal position on our initial hit to conduct fragment exchange with known type-II kinase inhibitors (sorafenib and AD80 [1]), we took into consideration (i) the docking poses and phenotypic results of these compounds and (ii) the synthetic accessibility and the novelty of the putative hybrid compounds, even if they do not dock optimally to our intended kinase targets. We focused on ***2***/***2-3*** due to: 1) their 1*H*-indole moiety docks uniquely into the DFG-pocket and with the potential to interact with the αC-helix glutamate (Fig 5C); 2) their 1*H*-indole-2-carboxamide moiety resembles the urea linker that is commonly found in type-II KIs such as sorafenib (Fig 6A; blue box); 3) the *N*-phenylcarboxamide moiety of ***2–3*** is a common linker between the hinge-binding and the DFG-pocket moieties of type-II KIs, e.g. imatinib (Fig 6A; grey box), while the *N*-(piperidin-4-yl)carboxamide moiety of ***2*** is not a common linker; 4) the docking pose of ***2***/***3***’s 1H-indole moiety overlaps with the trifluoromethylphenyl moiety of sorafenib/AD80. Based on these four criteria, we chose to perform a fragment exchange at the carboxamide position to combine the 1*H*-indole-2-carboxamide moiety of ***2***/***2-3*** with the hinge-binding moiety of sorafenib and of AD80, a multi-kinase inhibitor that has shown promise in MTC treatment [1], to create ***3*** and ***4***, respectively (Fig 6B).

Oral administration of ***3*** and ***4*** to *ptc>dRet*^*M955T*^ flies demonstrated that the efficacy of ***4*** was low with only 3% rescue, while ***3*** demonstrated strongly improved efficacy with 15% rescue (Fig 6C; *P* < 0.05); this level of rescue was significantly higher than the parent compound ***2/2-3*** or sorafenib. Additionally, ***3*** suppressed the invasion/migration of dRet^M955T^-expressing cells in the wing epithelium (Fig 6D), further confirming its efficacy against dRet^M955T^-mediated phenotypes. The kinase inhibition profile of ***3*** (Table 3) resembles that of the parent compound ***2–3*** (Table 2) with at least two notable exceptions: ***3*** inhibits CSF1R, PDGFRB, and FLT3, receptor tyrosine kinases and orthologs of *Drosophila* Pvr that activate the Ras/ERK signaling pathway [25] and play key roles in SRC activation and tumor progression; ***3*** inhibits Aurora kinases AURKB and AURKC (*Drosophila* ortholog aurA or aurB). Of note, although ***4*** did not improve the viability of *ptc>dRet*^*M955T*^ flies, it shares chemical similarity to several known type-I½ kinase inhibitors that have the common adenine moiety and a related indole moiety. This group of inhibitors was shown to inhibit other related kinases, increasing our confidence in the relevance of this chemical space for kinase pathway modulation [26].

**Table 3:** Kinase inhibition profile of compound 3 at 50 μM.

Kinase	% Inhib.	Kinase	% Inhib.
ABL1	0	mTOR	4
AURKA	15	**PDGFRB**	**98**
**AURKB**	**88**	RET	29
AURKC	**91**	RET (M918T)	26
**BRAF**	**4**	RET (V804L)	46
**CSF1R**	**95**	S6K1	0
FGFR	0	SRC	4
**FLT3**	**80**	TTK	27

Bold, inhibited by more than 40%.

## Discussion

### Integrated discovery pipeline

This study demonstrates the utility of an integrated platform that combines *Drosophila* genetics, computational structural biology, and synthetic chemistry to enrich for the discovery of useful chemical tools in an established *Drosophila* MTC model (Fig 2). We have previously shown that *Drosophila* can provide a unique entry point for drug development by capturing subtle structural changes in lead compounds that are often missed by cellular or biochemical assays [15]. Here we refine this approach by iteratively combining experimental testing with computational modeling. A key strength of the integrated approach is its ability to rapidly derive candidates from a large, purchasable chemical library via virtual screening to test chemically unique compounds with our fly models in a cost-effective manner. This platform allowed us to quickly confirm the *in situ* relevance of active chemotypes through iterations of computational modeling, synthetic chemistry, and phenotypic testing in the fly. We expect this integrated pipeline is generally applicable to kinase networks associated with other diseases [7].

### DFG-out modeling approach

DFGmodel is a recently developed computational tool that generates models of kinases in their inactive, DFG-out conformation for rational design of type-II KIs [10]. In a recent study, models generated by DFGmodel were used to guide the optimization of the drug sorafenib to better target a new disease space [15]. Here, we demonstrate a successful application of DFGmodel to explore compounds that are not appreciated as kinase inhibitors. For each kinase target, DFGmodel uses multiple experimentally determined structures as modeling templates and generates multiple homology models. Thus, this method samples a large fraction of the DFG-out conformational space during the model construction, which enables us to account for the flexibility of the binding site during virtual screening [27]. Notably, DFG-out models capture key features that are important for protein-ligand interactions in multiple kinases simultaneously, providing a framework for rationalizing activity of known inhibitors and developing unique KIs that target a signaling pathway. For example, our results suggest that the electrostatic potential within the DFG-pocket is a key feature for inhibitor selectivity: ERK has an inverse electrostatic potential in the DFG-pocket than that of our target kinases RET and BRAF (Fig 3), which may explain the insensitivity of ERK toward inhibitors such as sorafenib.

### Identification of biologically active compounds

Although used in the clinics for MTC, sorafenib (and its close analog regorafenib) show limited efficacy in MTC patients; this poor activity is mirrored in the *ptc>dRet*^*M955T*^ fly model, which was rescued 3–4% at 200 μM [15]. Despite considerable academic and industry effort, the known chemical space of kinase inhibitors is limited [7]. For example, sorafenib and regorafenib differ in only one non-hydrogen atom. Through structure-based virtual screening against multiple kinase targets in the MTC pathway, we discovered chemically unique compounds (S2 Table) with an ability to rescue *ptc>dRet*^*M955T*^ viability that is similar to sorafenib, an FDA-approved KI (Figs 4 and 5).

Importantly, our data indicates that these compounds act through key cancer networks. For example, compounds ***1***, ***2***, ***2–3*** and ***3*** all have shown the ability to suppress invasion of *ptc>dRet*^*M955T*^ cells in the wing epithelium. Previous work demonstrated that *dRet*^*M955T*^-mediated wing cell invasion is controlled by SRC [15, 28], which acts by controlling E-cadherin and Matrix Metalloproteases (MMPs). Of note, ***1***, ***2***, ***2–3*** and ***3*** each demonstrated significant activity against orthologs of *Drosophila* Pvr, a key regulator of Src: all show significant activity against human FLT3, while ***3*** shows additional activity against Pvr orthologs CSF1R and PDGFRB. In addition to being orthologs of Pvr, FLT3, CSF1R, and PDGFRB similarly can activate SRC [29]. We propose that this activity against regulators of SRC account for the ability of ***1***, ***2***, ***2–3*** and ***3*** to suppress invasion, a key first step in tumor metastasis. Other activities, for example, ***3***’s inhibition of Aurora kinases—required for proliferation during tumor progression [30]—likely also contributes. Indeed, AURK inhibitors are known to be active against MTC [31, 32] and synergy between AURKs and FLT3 is currently being explored clinically through a number of dual-AURKB/FLT3 inhibitors [33, 34].

### Recombination of building blocks for future inhibitors

Although the new tool compounds ***1*** and ***2*** are not themselves sufficiently potent to serve as therapeutic leads, they reveal diverse fragment-like pharmacophores that serve as starting points for an exploration of new chemical space. These pharmacophores can be further optimized by combining with well-developed chemotypes that are known to interact with kinase binding sites (*e*.*g*., the hinge binding region) to form more efficacious chemical probes [35]; this provides a key second step towards building effective compounds. For example, ***2*** and ***2–3*** include an 1*H*-indole moiety capable of occupying the DFG-pocket of protein kinases from different families and a carboxamide group commonly found in type-II KIs (Fig 6A). Guided by the docking poses of these compounds, the 1*H*-indole-2-carboxamide group was combined with an optimized hinge-binding moiety from sorafenib, to form a significantly more efficacious compound (i.e., ***3***). As indicated in the kinase inhibition profile of ***3*** (Table 3), it shares part of the target set of its constituents ***2*** and ***2–3***.

Despite its promise, our approach has several limitations. The computational modeling does not take into account conformational changes modulated by inter- or intra-molecular interactions between the kinase domain and binding partners (e.g., SH2/SH3 domains, scaffolding proteins), as well as the differential propensity among kinase domains to adopt DFG-out states [36]. This weakness is partly mitigated through careful template selection for model building as well as docking of the small molecules to multiple models representing different conformers. Second, although kinases are closely conserved between humans and *Drosophila*, fly models have some differences with human disease networks including lacking an adaptive immune system. They lack some relevant target organs (e.g., thyroid, breast, prostate, pancreas); in these cases we focused on developing eye and wing epithelia, which provide ‘generic’ polarized epithelia that can give biological and pharmacological results in *Drosophila* that have proven translatable to mammals as we previously reported [1, 15, 37–39]. Despite these limitations, flies offer a useful genetic and pharmacological toolkit which can facilitate drug development for cancer as we show in this study. Future studies will include testing the compounds discovered in this study on mammalian models including human MTC cell lines and mouse xenografts [15].

In summary, we demonstrate the potential of combining chemical modeling with *Drosophila* genetics to rapidly and efficiently explore novel chemical space. This provides an accessible and cost-effective platform that can be applied to a broad palette of diseases that can be modeled in *Drosophila*. Combining the strengths of these two high-throughput approaches opens the opportunity to develop novel tool and lead compounds that are effective in the context of the whole animal.

## Materials and methods

### DFG-out models

Models of kinase targets (human RET, SRC, BRAF, S6K1) in the DFG-out conformation were generated using DFGmodel [10]. Briefly, the method takes a DFG-in structure or the sequence of the protein kinase as input. DFG-model relies on a manually curated alignment between the target kinase and multiple structures representing unique DFG-out conformations. It calls on the structure-based sequence alignment function of T_COFFEE/Expresso [40] v11.00.8 to perform sequence alignment of the kinase catalytic domain to the templates, followed by the multi-template function of MODELLER [41] v9.14 to generate 50 homology models covering a range of conformations. For each kinase 10 DFG-out models with largest binding site volume, as calculated by POVME [42] v2.0, were used for further study. These ensembles of DFG-out models of our targets BRAF, p70-S6K, RET, and SRC have been evaluated and confirmed to enrich known type-II inhibitors over non-ligands derived from kinase-inhibitor complexes found in PDB in our previous study [10]. The area-under-curve (AUC) of virtual screening performance of our targets BRAF, RET, and SRC DFG-out models are 87.7, 82.8, and 76.8, respectively, which correspond to at least 4- to 5-fold increase in the enrichment accuracy over randomly selected ligands in a known sample set [43–45]. This ensemble of models provides an approximation of the binding site flexibility, as well as optimizes the binding site for protein-ligand complementarity and structure-based virtual screening [11, 27, 43].

### Virtual screening

Initial virtual screening utilized the ZINC12 [22] “available now” lead-like chemical library (downloaded in 2013, 2.2 million compounds). A maximum of 300 conformers for each compound were generated with OMEGA. Each conformer was docked with FRED using the default settings [40][41]. The top scoring pose was used for further analysis. For each of the four targeted kinases (RET, BRAF, SRC, p70-S6K), the ensemble of 10 DFG-out models was used for screening. Each compound was docked against 10 models of each kinase target, resulting in 10 docking poses per each kinase-compound pair. The results were filtered by selecting compounds that rank in the top 10% in at least five models per kinase-compound pair, represented by the best scoring pose. This filtering process was done for all four targets. Next, compounds that scored in the top 25% in at least 3 of 4 the kinase ensemble models were collated into a final set of 247 compounds. These consensus best-scoring ligands, representing 0.0112% of the library, were visually inspected to remove molecules with energetically unfavorable or strained conformations, or with reactive functional groups that may interfere with assays [46], which are commonly observed in large virtual screenings. Eight compounds, all top-100 ranking among 247 candidates (S1 Table), were selected based on their interactions with the models (DFG-pocket occupancy, hydrogen-bond to conserved amino acids, etc) and chemical novelty and were purchased for *Drosophila* viability screening. Analogs ***1–1***, ***1–2***, ***2–1 to 2–5***, and others (S2 Table) were identified based on the structure of compounds ***1*** and ***2*** through the chemical similarity search function available in ZINC15 [8] and SciFinder using the default setting and Tanimoto coefficient above 70% similarity. These compounds are commercially available through vendors such as ChemBridge and Enamine.

### Chemical methods

For synthetic procedures and characterization data related to compounds ***1***, ***3***, and ***4***, please see supplementary materials.

### Kinase profiling of compounds

Kinase inhibition profile of the compounds was assessed at 50 μM through commercially available kinase profiling services (DiscoverX).

### Drosophila stocks

Human orthologs of *Drosophila* genes were predicted by DIOPT (http://www.flyrnai.org/cgi-bin/DRSC_orthologs.pl). The multiple endocrine neoplasia (MEN) type 2B mutant form of *Drosophila* Ret carries the M955T mutation (dRet^M955T^), which corresponds to the M918T mutation found in human MTC patients. The *ptc-gal4*, *UAS-GFP*; *UAS-dRet*^*M955T*^/*SM5(tub-gal80)-TM6B* transgenic flies were prepared according to standard protocols [15]. In these flies, the *tubulin* promoter drives GAL80, a suppressor of GAL4, to repress dRet^M955T^ expression. We crossed them with *w*^*-*^ flies to obtain *ptc*>*dRet*^*M955T*^ flies that lost *GAL80* allele, which derepressed dRet^M955T^ expression (S1A Fig). Transgenic *ptc>Ret*^*M955T*^ flies were calibrated to have 0% survival when raised at 25°C, which allowed for drug screening (S1B Fig).

### Chemical genetic screening in flies

We employed dominant modifier screening [15] using the *ptc-gal4*, *UAS-GFP*; *UAS-dRet*^*M955T*^/*SM5(tub-gal80)-TM6B* to screen for fly kinase genes that affected the *dRet*^*M955T*^-induced lethality in flies when heterozygous (*ptc>Ret*^*M955T*^;*kinase*^*-/+*^). Genes that improved or reduced survival of *ptc>dRet*^*M955T*^ flies when heterozygous were designated as genetic ‘suppressors’ or ‘enhancers’, respectively (Fig 1B). Suppressors are candidate therapeutic targets that when inhibited may reduce tumor progression.

Stock solutions of the test compounds were created by dissolving the compound in DMSO at the maximum concentration. The stock solutions were diluted by 1,000-fold or more and mixed with semi-defined fly medium (Bloomington Drosophila Stock Center) to make drug-infused food (0.1% final DMSO concentration; maximum tolerable dose in flies). Approximately 100 *ptc>dRet*^*M955T*^ embryos alongside with wild-type (*+;+/SM5*_*tubgal80*_*-TM6B*) flies were raised until adulthood on drug-infused food for 13 days at 25°C. The numbers of empty pupal cases (*P* in S1B Fig) and that of surviving adults (*A*) were used to determine percentage of viability, while their body size, which is affected by food intake, temperature, and humidity, were compared to vehicle-treated groups to standardize the experimental conditions. For toxicity test, the viability of the wild-type flies raised alongside with the *ptc>dRet*^*M955T*^ flies in the same vials was examined. None of the tested compounds show detrimental effect in the control flies, showing > 90% viability at any doses tested for the compounds equivalent to vehicle.

### Wing discs cell migration/invasion assays

Third-instar *ptc>dRet*^*M955T*^ larvae were dissected, and developing wing discs were collected, fixed with 4% paraformaldehyde in PBS, and whole-mounted. At least 10 wing discs were analyzed for each treatment. Invasive GFP-labeled dRet^M955T^-expressing cells were visualized by their green pseudocolor under a confocal microscope. The apical and the virtual z-series views of the wing disc were examined to identify abnormal tissue growth and dRet^M955T^-expressing cells migrating beyond the *ptc* domain boundary.

## Supporting information

S1 Fig **(A)** Preparation of transgenic *ptc*>*dRet*^*M955T*^ flies for chemical genetic screening [3]. **(B)** Determination of compound efficacy in a fly-based chemical genetic screening. The numbers of empty pupal cases (*P*) and surviving adult (*A*) are used to determine viability.
(TIF)

S2 Fig DFG-pocket of various protein kinases. The left panels show the DFG-pocket (colored volume) with the docking pose of ***1***. The right panels show the electrostatic potential on the surface of DFG-pocket (blue, positive; red, negative).
(TIF)

S3 Fig Common interactions in type-II inhibitor binding site. Type-II kinase inhibitors are modular. They are composed of a hinge-binding moiety and a spacer group, followed by a linker that forms hydrogen bonds with the conserved glutamate residue on the αC-helix, as well as a hydrophobic “cap” group that docks into the DFG-pocket. Key elements in type-II inhibitor/kinase interactions include **(A)** Hydrogen bonds with “hinge” amide backbone. **(B)** π-π stacking with DFG-Phe. **(C)** Hydrogen bonds with αC-helix glutamate. **(D)** Hydrogen bond with DFG-Asp amide backbone. **(E)** van der Waals interactions in DFG-pocket.
(TIF)

S1 Table Compounds identified from the ZINC12 lead-like dataset and their ranking in the 247-compound consensus virtual screening result of the four kinase models. (DOCX)

S2 Table Initial hits, their active purchasable analogs, and synthesized analogs. (DOCX)

S1 Text Compound synthesis and characterization. (DOCX)
