# Supplementary figures and images for: Integrated computational and Drosophila cancer model platform captures previously unappreciated chemicals perturbing a kinase network
Source: PLoS Comput Biol. 2019 Apr 26;15(4):e1006878. doi: 10.1371/journal.pcbi.1006878 (PMC6506148; doi:10.1371/journal.pcbi.1006878)

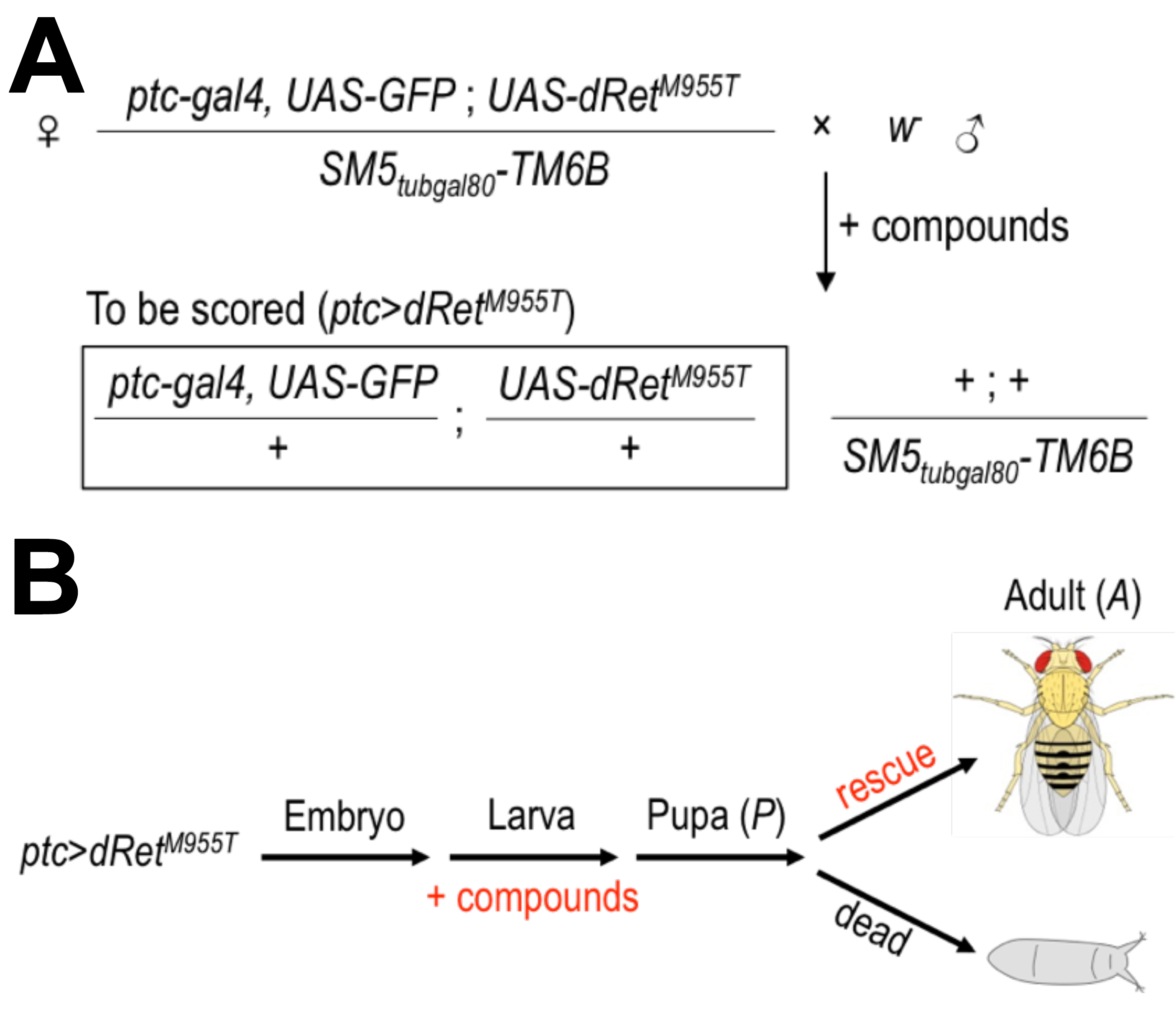

Supplement: S1 Fig — (A) Preparation of transgenic ptc>dRetM955T flies for chemical genetic screening [3]. (B) Determination of compound efficacy in a fly-based chemical genetic screening. The numbers of empty pupal cases (P) and surviving adult (A) are used to determine viability. (TIF) [file pcbi.1006878.s001.tif]

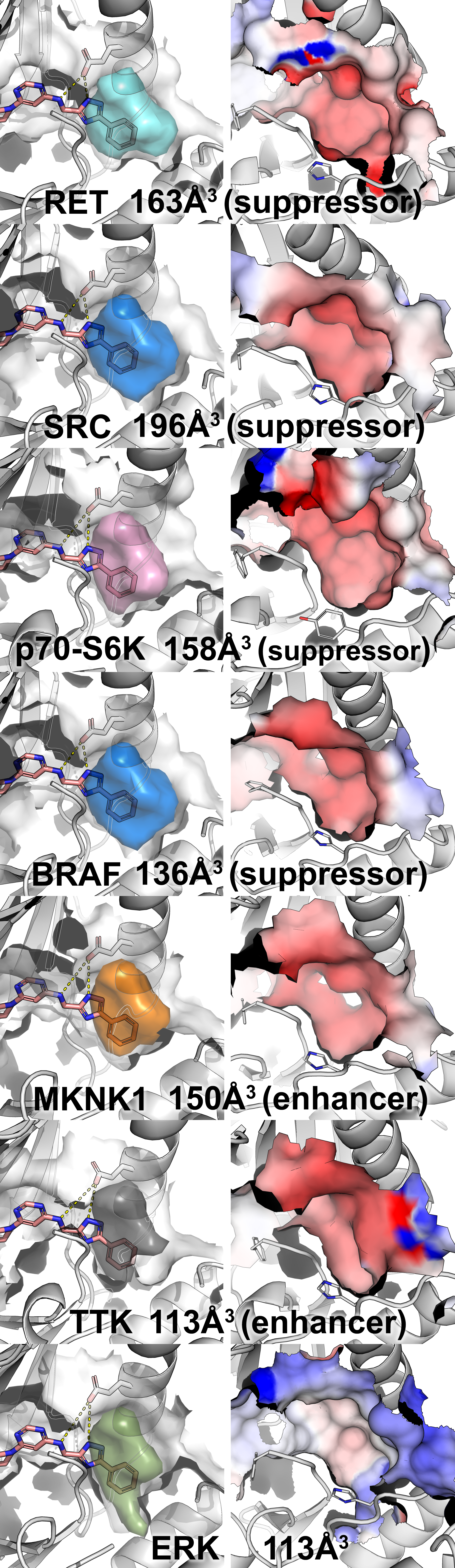

Supplement: S2 Fig — The left panels show the DFG-pocket (colored volume) with the docking pose of 1. The right panels show the electrostatic potential on the surface of DFG-pocket (blue, positive; red, negative). (TIF) [file pcbi.1006878.s002.tif]

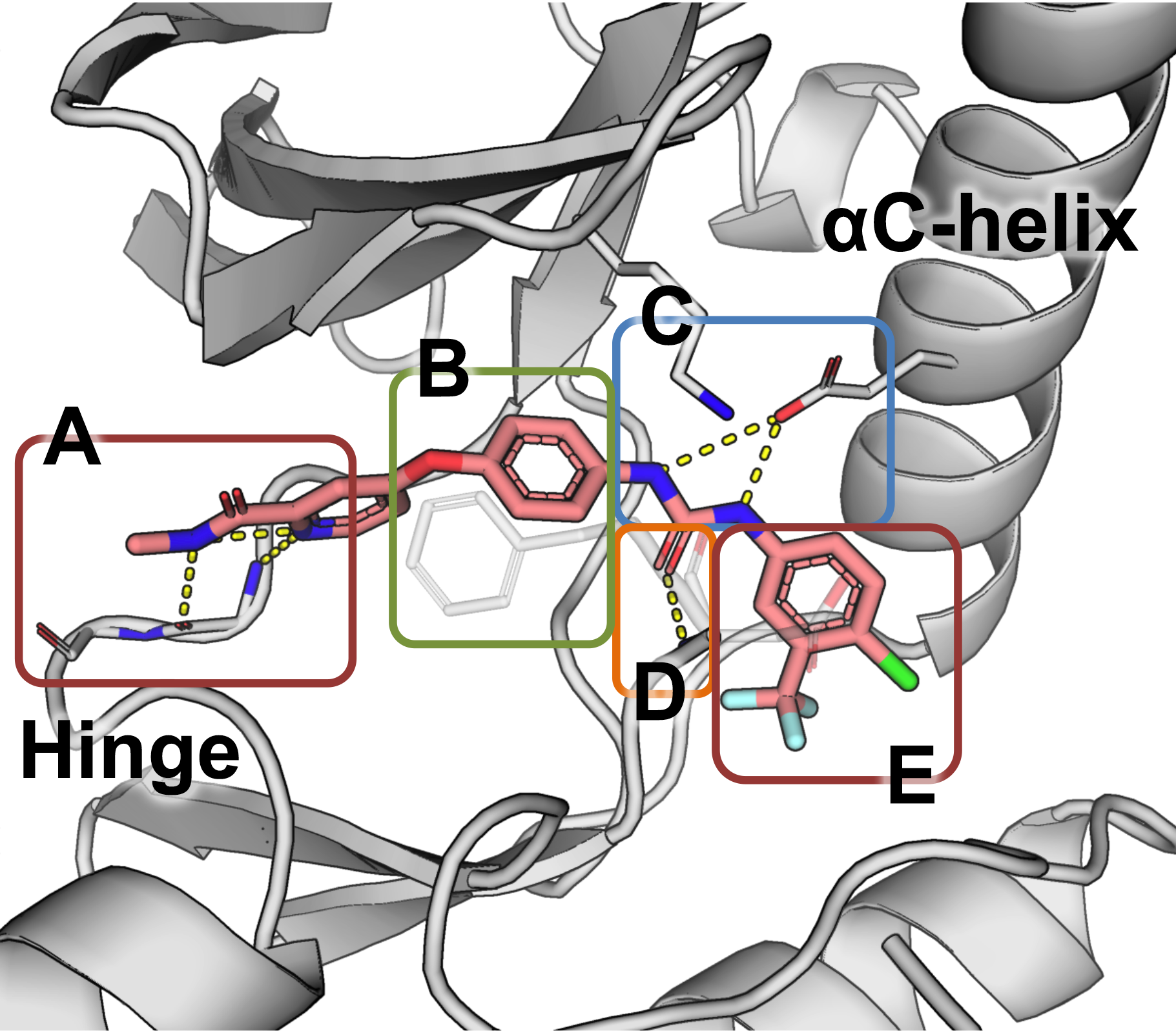

Supplement: S3 Fig — Type-II kinase inhibitors are modular. They are composed of a hinge-binding moiety and a spacer group, followed by a linker that forms hydrogen bonds with the conserved glutamate residue on the αC-helix, as well as a hydrophobic “cap” group that docks into the DFG-pocket. Key elements in type-II inhibitor/kinase interactions include (A) Hydrogen bonds with “hinge” amide backbone. (B) π-π stacking with DFG-Phe. (C) Hydrogen bonds with αC-helix glutamate. (D) Hydrogen bond with DFG-Asp amide backbone. (E) van der Waals interactions in DFG-pocket. (TIF) [file pcbi.1006878.s003.tif]
