# Supplementary material for: Integrated computational and Drosophila cancer model platform captures previously unappreciated chemicals perturbing a kinase network
Source: PLoS Comput Biol. 2019 Apr 26;15(4):e1006878. doi: 10.1371/journal.pcbi.1006878 (PMC6506148; doi:10.1371/journal.pcbi.1006878)
Supplement: S1 Table — (DOCX) [file pcbi.1006878.s004.docx]

| S1 Table. Compounds identified from the ZINC12 lead-like dataset and their ranking in the 247-compound consensus virtual screening result of the four kinase models. | | | | | | |
| --- | --- | --- | --- | --- | --- | --- |
| Name | ZINC ID | Rank |  |  | ZINC ID | Rank |
| *1* | 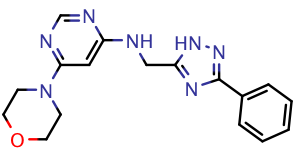  ZINC65373673 | 12 |  |  | 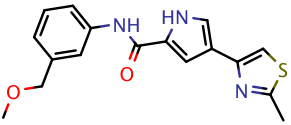  ZINC25335400 | 20 |
| *2* | 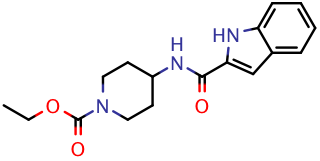  ZINC23798680 | 87 |  |  | 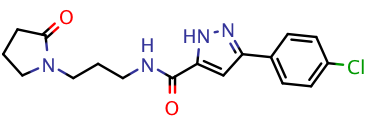  ZINC32645153 | 42 |
|  | 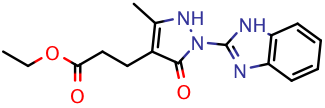  ZINC9060186 | 33 |  |  | 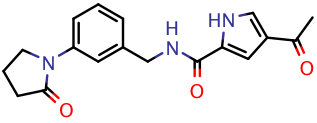  ZINC44935144 | 67 |
|  | 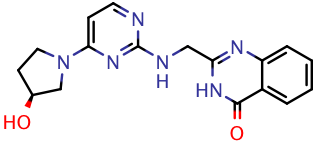  ZINC65455152 | 1 |  |  | 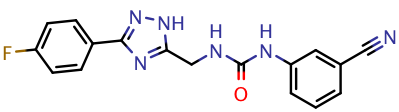  ZINC69621870 | 70 |
|  |  |  |  |  |  |  |
|  |  |  |  |  |  |  |
| Inactive analogs of *1* and *2* based on chemical similarity | | | | | |  |
|  | 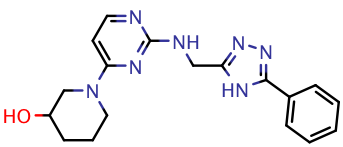  Chembridge 78510425 |  |  |  | 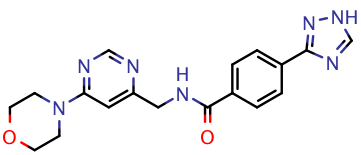  Chembridge 58946452 |  |
|  | 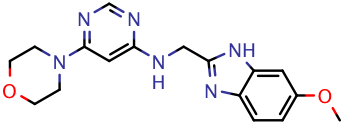  Chembridge 38168496 |  |  |  |  |  |
