# Supplementary material for: Integrated computational and Drosophila cancer model platform captures previously unappreciated chemicals perturbing a kinase network
Source: PLoS Comput Biol. 2019 Apr 26;15(4):e1006878. doi: 10.1371/journal.pcbi.1006878 (PMC6506148; doi:10.1371/journal.pcbi.1006878)
Supplement: S2 Table — (DOCX) [file pcbi.1006878.s005.docx]

| **S2 Table.** Initial hits, their active purchasable analogs, and synthesized analogs. | | | | | | |  |
| --- | --- | --- | --- | --- | --- | --- | --- |
| Name | ZINC ID | MW |  | Name | ZINC ID | MW |  |
| Sorafenib | 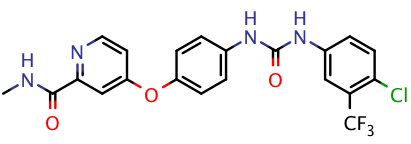  ZINC01493878 | 465 |  | ***2-1*** | 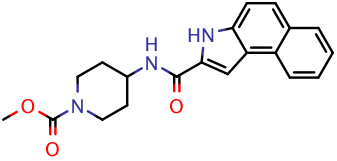  ZINC57448002 | 351 |  |
| ***1*** | 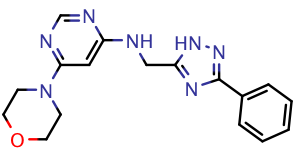  ZINC65373673 | 337 |  | ***2-2*** | 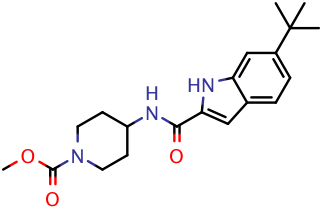  ZINC57448228 | 357 |  |
| ***2*** | 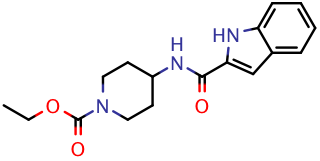  ZINC23798680 | 315 |  | ***2-3*** | 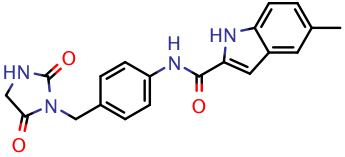  ZINC29176427 | 362 |  |
| ***1-1*** | 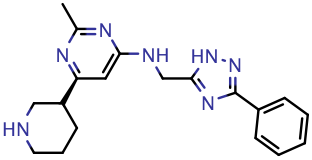  ZINC39500466 | 349 |  | ***2-4*** | 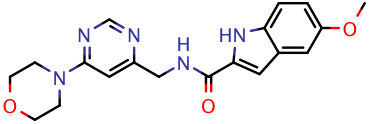  ZINC72436343 | 367 |  |
| ***1-2*** | 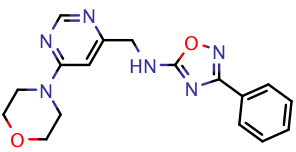  ZINC72480555 | 338 |  | ***2-5*** | 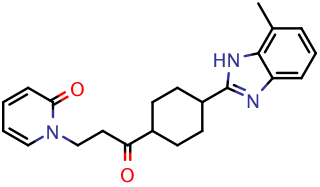  ZINC82098761 | 364 |  |
| ***3*** | 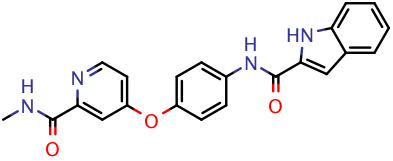 | 386 |  | ***4*** | 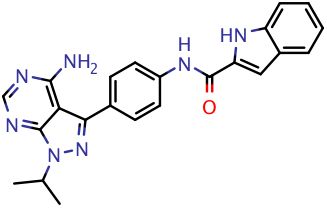 | 411 |  |

MW, molecular weight (g/mol).
