# Supplementary material for: Integrated computational and Drosophila cancer model platform captures previously unappreciated chemicals perturbing a kinase network
Source: PLoS Comput Biol. 2019 Apr 26;15(4):e1006878. doi: 10.1371/journal.pcbi.1006878 (PMC6506148; doi:10.1371/journal.pcbi.1006878)
Supplement: S1 Text — (DOCX) [file pcbi.1006878.s006.docx]

**Supporting Information**

**Integrated *in silico* and *Drosophila* cancer model platform captures previously unappreciated chemicals perturbing a kinase network**

Peter Man-Un Ung^1^†, Masahiro Sonoshita^2^†, Alex P. Scopton^3^, Arvin C. Dar^3^, Ross L. Cagan^2^*, Avner Schlessinger^1^*

*^1^Department of Pharmacological Sciences, Icahn School of Medicine at Mount Sinai, New York, NY, 10029, USA*

*^2^Department of Cell, Developmental and Regenerative Biology, Icahn School of Medicine at Mount Sinai, New York, NY 10029, USA*

*^3^Department of Oncological Sciences, Icahn School of Medicine at Mount Sinai, New York, NY 10029, USA*

* Corresponding authors. E-mail:

[ross.cagan@mssm.edu](mailto:ross.cagan@mssm.edu) [avner.schlessinger@mssm.edu](mailto:avner.schlessinger@mssm.edu)

† These authors contributed equally to this work.

***Table of Contents***

Section Page

General Chemical Methods: S1 Text S2

Synthesis of Hybrid Molecule ***1*** S3

Synthesis of Hybrid Molecule ***3*** S4

Synthesis of Intermediates ***3a*** and ***3b*** in route to ***3*** S4 – S5

Synthesis of Hybrid Molecule ***4*** S6

Synthesis of Intermediates ***4a*** and ***4b*** in route to ***4*** S6 – S7

LC-MS and ^1^H NMR Spectra for ***1***, ***3***, and ***4*** S8 – S13

References S14

**S1 Text**

**General Chemical Methods.** All solvents were purchased from Sigma-Aldrich and were used as received; anhydrous solvents were used for chemical reactions, and HPLC grade solvents were used for aqueous work-ups, recrystallizations and chromatography. Other reagents were purchased from various vendors and were used as received. Reactions were run as described in the individual procedures using standard double manifold and syringe techniques. Glassware was dried by baking in an oven at 130 °C for 12h prior to use, or was flame-dried. Vacuum filtrations were carried out using a house vacuum line (~100 torr). In the individual procedures, the phrases “concentration under vacuum” and “concentrated to dryness” mean that solvent was removed on a rotary evaporator using a diaphragm pump (with an automatic vacuum regulator) and remaining traces of volatiles were removed on a high-vacuum (<1 torr) oil pump. Unless specified otherwise, the term “flask” refers to the round-bottomed variety. Reactions were monitored by TLC using EMD silica gel 60 F_254_ (250 µm) glass-backed plates (visualized by UV fluorescence quenching and stained with basic KMnO_4_ solution) and by liquid chromatography-tandem mass spectrometry (LC-MS). Analysis by reverse-phase LC-MS was carried out on a Waters Acquity I-Class UPLC system, with a C18 column (2.1 x 30 mm; 1.7 µm particle size), heated at 50 °C, eluting at 0.6 mL/min, and using a 3 min linear gradient method with a mobile phase consisting of water/acetonitrile (0.1% v/v formic acid added to each): 95:5→1:99(0-2.5 min), then 1:99(2.5-3 min). Sample runs were monitored using alternating positive/negative electrospray ionization (50-1000 amu) and UV detection at 254 nm. Dimensions of plugs, pads and columns for filtration or flash chromatography are reported as: ((diameter x length) cm). The 5¾ inch pipets (4 mL) used for filtration and micro scale flash chromatography were purchased from Fisher Scientific (product number 22-378-893). Automated preparative normal- and reverse-phase chromatography was carried out with an Interchim PuriFlash 450 purification system with a diode array detector (runs were monitored at 220-400 nm). Pre-packed silica gel cartridges (12, 25 and 40 g; 15 µm particle size) were employed for normal-phase (silica gel) chromatography, eluting at 20-30 mL/min. For reverse-phase chromatography a C18 column (30 x 150 mm; 5 µm particle size) was used, eluting at 15-20 mL/min with a pressure limit of 50 bar. Carbon-decoupled ^1^H NMR spectra were recorded at 400 MHz on a Bruker spectrometer and are reported in ppm using the residual solvent signal (dimethylsulfoxide-d6 = 2.50 ppm) as an internal standard. Data are reported as: [(shift), [(s=singlet, d=doublet, dd=doublet of doublets, ddd=doublet of a doublet of doublets, t=triplet, dt=doublet of triplets, q=quartet, sept=septet, m=multiplet, br=broad, ap=apparent), (*J*=coupling constant in Hz), (integration)]]. Proton-decoupled ^13^C NMR spectra were recorded at 100 MHz on a Bruker spectrometer and are reported in ppm using the residual solvent signal (dimethylsulfoxide-d_6_ = 39.5 ppm) as an internal standard.

**Morpholino-*N*-((5-phenyl-4*H*-1,2,4-triazol-3-yl)methyl)pyrimidin-4-amine (*1*).** A 15 mL pressure tube was charged with 4-(6-chloropyrimidin-4-yl)morpholine (80.8 mg, 0.405 mmol), (5-phenyl-4*H*-1,2,4-triazol-3-yl)methanamine dihydrochloride (100 mg, 0.405 mmol), n-BuOH (1.5 mL) and Et_3_N (230 µL, 1.65 mmol). The tube was sealed with a threaded Teflon plug and the mixture was heated at 150 °C for 18 h. After cooling to room temperature, the tube was opened, the reaction was diluted with water (20 mL) and extracted (3 x 20 mL) with a mixture of CH_2_Cl_2_/MeOH (95:5). The organic extracts were pooled, dried (Na_2_SO_4_), filtered and concentrated under vacuum. The remaining material was purified by silica gel chromatography (25 g cartridge), eluting at 25 mL/min and using a linear gradient of CH_2_Cl_2_/MeOH: 100:0→85:15 over 22 column volumes. Obtained 34.0 mg (40%) of the title compound as a white solid: **^1^H NMR** (400 MHz, DMSO-d_6_) δ ppm 13.93 (br s, 1H), 8.04 (s, 1H), 7.97 (dd, *J*=8.2, 1.3 Hz, 2H), 7.37 - 7.54 (m, 3H), 7.29 (br s, 1H), 5.78 (s, 1H), 4.57 (br s, 2H), 3.60 - 3.68 (m, 4H), 3.36 - 3.44 (m, 4H); **LC-MS** (ESI+) *m/z*: [M+H]^+^ Calcd for C_17_H_20_N_7_O 338.2, found 338.3.

***
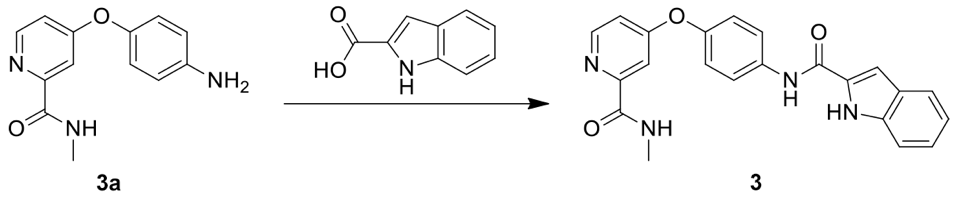
***

***N*-(4-((2-(Methylcarbamoyl)pyridin-4-yl)oxy)phenyl)-1*H*-indole-2-carboxamide (*3*).**

To a solution of 1*H*-indole-2-carboxylic acid (58.3 mg, 0.362 mmol), HATU (140 mg, 0.368 mmol) and DMF (1 mL), in an 8 mL vial, was added DIPEA (70.0 μL, 0.402 mmol) dropwise over 1 min. The solution was stirred for 30 min and then ***3a*** (80.0 mg, 0.329 mmol) was added in one portion. The reaction was blanketed with Ar, the vial was sealed with a screwcap and stirring was continued for 12 h. The reaction was diluted with half saturated NaHCO_3_ solution (20 mL) and extracted with CH_2_Cl_2_ (3 x 20 mL). The organic extracts were pooled, dried (Na_2_SO_4_), filtered and concentrated to dryness. The remaining residue was purified by silica gel chromatography (12 g cartridge), eluting at 20 mL/min and using a linear gradient of hexanes/EtOAc: 100:0→0:100 over 20 column volumes. Obtained 50.0 mg (39%) of the title compound as a white solid: **^1^H NMR** (400 MHz, DMSO-d_6_) δ ppm 11.78 (d, *J*=1.5 Hz, 1H), 10.36 (s, 1H), 8.78 (q, *J*=4.7 Hz, 1H), 8.52 (d, *J*=5.6 Hz, 1H), 7.95 (d, *J*=9.0 Hz, 2H), 7.69 (d, *J*=8.1 Hz, 1H), 7.48 (dd, *J*=8.2, 0.9 Hz, 1H), 7.43 - 7.46 (m, 1H), 7.42 (d, *J*=2.4 Hz, 1H), 7.20 - 7.28 (m, 3H), 7.17 (dd, *J*=5.5, 2.6 Hz, 1H), 7.08 (ddd, *J*=7.9, 7.0, 1.0 Hz, 1H), 2.79 (d, *J*=4.9 Hz, 3H); **^13^C NMR** (100 MHz, DMSO-d_6_) δ ppm 165.8, 163.8, 159.8, 152.5, 150.4, 148.7, 136.9, 136.8, 131.3, 127.0, 123.8, 121.9, 121.8, 121.3, 120.0, 114.1, 112.4, 108.8, 104.0, 26.0; **LC-MS** (ESI+) *m/z*: [M+H]^+^ Calcd for C_22_H_19_N_4_O_3_ 387.1, found 387.2.

**
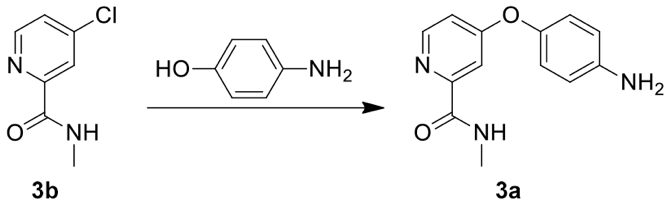
**

**4-(4-Aminophenoxy)-*N*-methylpicolinamide (*3a*).**

A two-necked 100 mL flask (equipped with an inlet adapter and septum) was flame-dried under vacuum and cooled under Ar. The flask was charged with 4-aminophenol (2.09 g, 19.2 mmol) and DMF (30 mL). To the stirred solution was added potassium *tert*-butoxide (2.14 g, 19.1 mmol) in portions over 1 min. The resulting light-brown mixture was stirred for 2 h, then 4-chloro-*N*-methylpicolinamide (***3b***; 2.17 g, 12.7 mmol) was added in one portion, and the reaction was heated at 80 °C for 4 h under a balloon of Ar. The reaction was allowed to cool to room temperature and then was poured into stirred ice-water (100 mL). Stirring was continued for 15 min and then the mixture was extracted with EtOAc (1 x 100 mL and 2 x 50 mL). The organic extracts were pooled, washed with 1 M KOH (3 x 50 mL), water (50 mL) and brine (2 x 50 mL), dried (Na_2_SO_4_) and filtered. Concentration under vacuum gave 3.23 g of an orange oil, which was purified by silica gel chromatography (40 g cartridge), eluting at 30 mL/min and using a linear gradient of hexanes/EtOAc: 100:0→0:100 over 35 column volumes. Obtained 2.69 g (87%) of the title compound as an off-white solid: **^1^H NMR** (400 MHz, DMSO-d_6_) δ ppm 8.73 (br q, *J*=4.6 Hz, 1H), 8.45 (d, *J*=5.6 Hz, 1H), 7.34 (d, *J*=2.5 Hz, 1H), 7.06 (dd, *J*=5.5, 2.6 Hz, 1H), 6.86 (d, *J*=8.8 Hz, 2H), 6.64 (d, *J*=8.8 Hz, 2H), 5.17 (s, 2H), 2.78 (d, *J*=4.9 Hz, 3H); **^13^C NMR** (100 MHz, DMSO-d_6_) δ ppm 166.8, 163.9, 152.3, 150.1, 146.9, 142.8, 121.6, 114.9, 113.7, 108.3, 26.0; **LC-MS** (ESI+) *m/z*: [M+H]^+^ Calcd for C_13_H_14_N_3_O_2_ 244.1, found 244.2; these data are in agreement with that previously reported[^2^](#_ENREF_2).

**
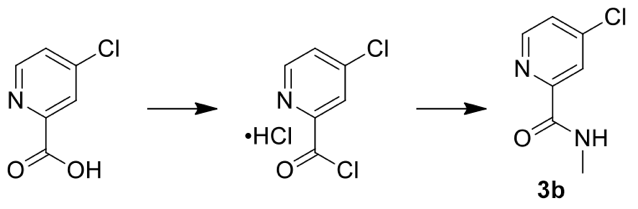
**

**4-Chloro-*N*-methylpicolinamide (*3b*).**

A flame-dried 250 mL flask, cooled under Ar, was charged with 4-chloropicolinic acid (10.0 g, 63.5 mmol), and THF (125 mL). The mixture was cooled to 0 °C and oxalyl chloride (6.70 mL, 79.2 mmol) was added dropwise over 5 min via syringe, followed by DMF (0.1 mL), which was added by syringe in one shot (**CAUTION:** rapid release of gas). After 30 min the reaction mixture was allowed to warm to room temperature and was stirred under a balloon of Ar for 15 h. The resulting brown solution was concentrated on a rotary-evaporator; a drying tube filled with KOH pellets was used to trap residual HCl. The remaining oil was concentrated to dryness from toluene (3 x 10 mL) and then was dried further under high vacuum to provide a solid. The crude 4-chloropicolinoyl chloride hydrochloride salt was placed under Ar and THF (50 mL) was added. The dark solution was cooled to 0 °C and methylamine (160 mL, 2.0 M solution in THF, 320 mmol) was added dropwise over 20 min via syringe. After 5 min the reaction was allowed to warm to room temperature and was stirred for 16 h. The reaction mixture was diluted with water (200 mL) and extracted with EtOAc (3 x 150 mL). The organic extracts were pooled, washed with water (100 mL) and brine (2 x 100 mL), dried (Na_2_SO_4_) and filtered. Concentration under vacuum gave ~11 g of a red-brown oil, which was purified by silica gel chromatography (40 g cartridge), eluting at 30 mL/min and using a linear gradient of hexanes/EtOAc: 100:0→0:100 over 30 column volumes. The appropriate fractions were pooled and concentrated to dryness. The remaining clear colorless oil (~10 g) was dissolved in a mixture of hexanes/CH_2_Cl_2_ (4:1; 150 mL) and allowed to stand at –20 °C for 12 h. The resulting precipitate was isolated by vacuum filtration, washed with hexanes (2 x 30 mL) and air-dried to yield 8.90 g (82%) of the title compound as a white solid: **^1^H NMR** (400 MHz, DMSO-d_6_) δ ppm 8.85 (br ap d, *J*=3.4 Hz, 1H), 8.62 (dd, *J*=5.3, 0.6 Hz, 1H), 8.01 (dd, *J*=2.2, 0.6 Hz, 1H), 7.75 (dd, *J*=5.3, 2.2 Hz, 1H), 2.82 (d, *J*=4.9 Hz, 3H); **^13^C NMR** (100 MHz, DMSO-d_6_) δ ppm 163.1, 151.8, 150.0, 144.5, 126.3, 121.8, 26.1; **LC-MS** (ESI+) *m/z*: [M+H]^+^ Calcd for C_7_H_8_ClN_2_O 171.0, found 171.1; these data are in agreement with that previously reported[^2^](#_ENREF_2).

***
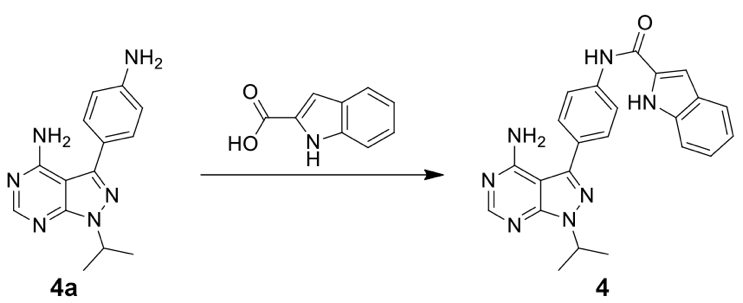
***

***N*-(4-(4-Amino-1-isopropyl-1*H*-pyrazolo[3,4-*d*]pyrimidin-3-yl)phenyl)-1*H*-indole-2-carboxamide (*4*).**

To a solution of 1*H*-indole-2-carboxylic acid (78.2 mg, 0.485 mmol), HATU (184 mg, 0.485 mmol) and DMF (2 mL), in an 8 mL vial, was added DIPEA (100 μL, 0.574 mmol) dropwise over 1 min. The solution was stirred for 30 min and then ***4a*** (100 mg, 0.373 mmol) was added in one portion. The reaction was blanketed with Ar, the vial was sealed with a screwcap and stirring was continued for 12 h. The solution was diluted with half saturated NaHCO_3_ solution (30 mL) and extracted with a mixture (95:5) of CH_2_Cl_2_/MeOH (3 x 30 mL). The organic extracts were pooled, dried (Na_2_SO_4_), filtered and concentrated to dryness. The remaining residue was purified by reverse-phase chromatography, eluting at 20 mL/min and using a linear gradient of H_2_O/MeCN: 90:10→0:100 over 30 minutes. Obtained 71 mg (46%) of the title compound as a white solid: **^1^H NMR** (400 MHz, DMSO-d_6_) δ ppm 11.78 (s, 1H), 10.39 (s, 1H), 8.24 (s, 1H), 8.02 (d, *J*=8.8 Hz, 2H), 7.65 - 7.73 (m, 3H), 7.46 - 7.51 (m, 2H), 7.24 (ddd, *J*=8.3, 7.1, 1.2 Hz, 1H), 7.08 (ddd, *J*=8.0, 7.0, 0.9 Hz, 1H), 6.74 (br s, 2H), 5.07 (sept, *J*=6.6 Hz, 1H), 1.50 (d, *J*=6.9 Hz, 6H); **^13^C NMR** (100 MHz, DMSO-d_6_) δ ppm 159.8, 158.1, 155.4, 153.3, 143.1, 139.4, 136.9, 131.3, 128.7, 128.2, 127.0, 123.9, 121.8, 120.3, 120.0, 112.4, 104.1, 97.5, 48.1, 21.8; **LC-MS** (ESI+) *m/z*: [M+H]^+^ Calcd for C_23_H_22_N_7_O 412.2, found 412.4.


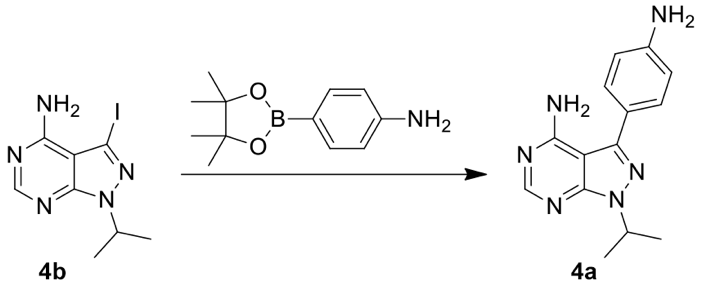


**3-(4-Aminophenyl)-1-isopropyl-1*H*-pyrazolo[3,4-*d*]pyrimidin-4-amine (*4a*).**

A 40 mL vial was charged with 3-iodo-1-isopropyl-1*H*-pyrazolo[3,4-*d*]pyrimidin-4-amine (***4b***; 618 mg, 2.04 mmol), 4-(4,4,5,5-tetramethyl-1,3,2-dioxaborolan-2-yl)aniline (581 mg, 2.65 mmol), Na_2_CO_3_ (650 mg, 6.13 mmol) and tetrakis(triphenylphosphine)palladium(0) (118 mg, 0.102 mmol), then 1,4-dioxane (16 mL) and water (4 mL) were added (both solvents were deoxygenated by sparging with Ar for 10 min). The headspace was purged with Ar, the vial was sealed with a screwcap and the reaction mixture was heated at 90 °C for 24 h. After the reaction had cooled to room temperature it was diluted with a mixture (95:5) of CH_2_Cl_2_/MeOH (15 mL) and water (5 mL), and then vacuum filtered through a pad (3 x 3 cm) of Celite; the pad was washed with CH_2_Cl_2_ (2 x 10 mL). The combined filtrates were transferred to a separatory funnel, diluted with brine (40 mL) and the layers were separated; the aqueous layer was extracted with CH_2_Cl_2_ (2 x 50 mL). The organic extracts were pooled, dried (Na_2_SO_4_), filtered and concentrated to dryness. The remaining semi-solid was purified by silica gel chromatography (25 g cartridge), eluting at 25 mL/min and using a linear gradient of CH_2_Cl_2_/MeOH: 100:0→90:10 over 30 column volumes. Obtained 520 mg (95%) of the title compound as an off-white solid: **^1^H NMR** (400 MHz, DMSO-d_6_) δ ppm 8.19 (s, 1H), 7.31 (d, *J*=8.6 Hz, 2H), 6.70 (d, *J*=8.6 Hz, 2H), 5.41 (br s, 2H), 5.01 (sept, *J*=6.7 Hz, 1H), 1.46 (d, *J*=6.6 Hz, 6H), the signal corresponding to the –NH_2_ group on the pyrimidine ring was not well resolved due to broadening into the baseline, but it appears to span the region 5.75 - 7.75 ppm; **LC-MS** (ESI+) *m/z*: [M+H]^+^ Calcd for C_14_H_17_N_6_ 269.2, found 269.4; these data are in agreement with that previously reported[^3^](#_ENREF_3).

**
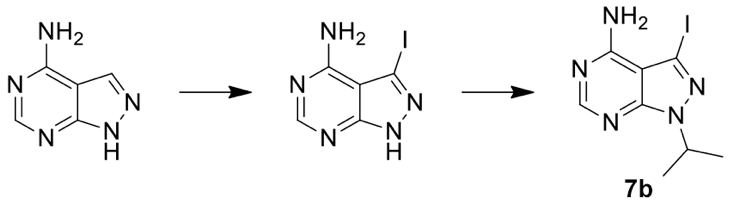
**

**3-Iodo-1-isopropyl-1*H*-pyrazolo[3,4-*d*]pyrimidin-4-amine (*4b*).**

A flame-dried 100 mL flask, cooled under Ar, was charged with 1*H*-pyrazolo[3,4-*d*]pyrimidin-4-amine (5.00 g, 37.0 mmol), *N*-iodosuccinimide (12.5 g, 55.6 mmol) and DMF (40 mL). The mixture was heated at 80 °C, under a balloon of Ar, for 22 h. The reaction mixture was allowed to cool to room temperature, diluted with water (40 mL) and stirred for 20 min. The solid was collected by vacuum filtration, washed with water (3 x 10 mL) and air-dried to provide 3-iodo-1*H*-pyrazolo[3,4-*d*]pyrimidin-4-amine (8.16 g, 31.3 mmol) as an off-white solid. The solid was added to a flame-dried 200 mL flask, under Ar, followed by oven-dried K_2_CO_3_ (5.19 g, 37.6 mmol) and DMF (60 mL). 2-Bromopropane (3.00 mL, 32.0 mmol) was added by syringe in a steady stream and the mixture was heated at 80 °C under a balloon of Ar for 18 h. The reaction was allowed to cool to room temperature, diluted with water (150 mL) and extracted with EtOAc (3 x 150 mL). The organic extracts were pooled, washed with water (2 x 100 mL) and brine (2 x 100 mL), dried (Na_2_SO_4_) and filtered. Concentration under vacuum gave an orange solid, which was recrystallized from MeOH (~100 mL). The solid was isolated by vacuum filtration, washed with EtOH (25 mL) and hexanes (2 x 25 mL), and then air-dried. Obtained 6.13 g (65% over two steps) of the title compound as white needles: **^1^H NMR** (400 MHz, DMSO-d_6_) δ ppm 8.19 (s, 1H), 7.19 (br s, 2H), 4.96 (sept, *J*=6.7 Hz, 1H), 1.42 (d, *J*=6.6 Hz, 6H); **LC-MS** (ESI+) *m/z*: [M+H]^+^ Calcd for C_8_H_11_IN_5_ 304.0, found 304.2; these data are in agreement with that previously reported[^3^](#_ENREF_3).


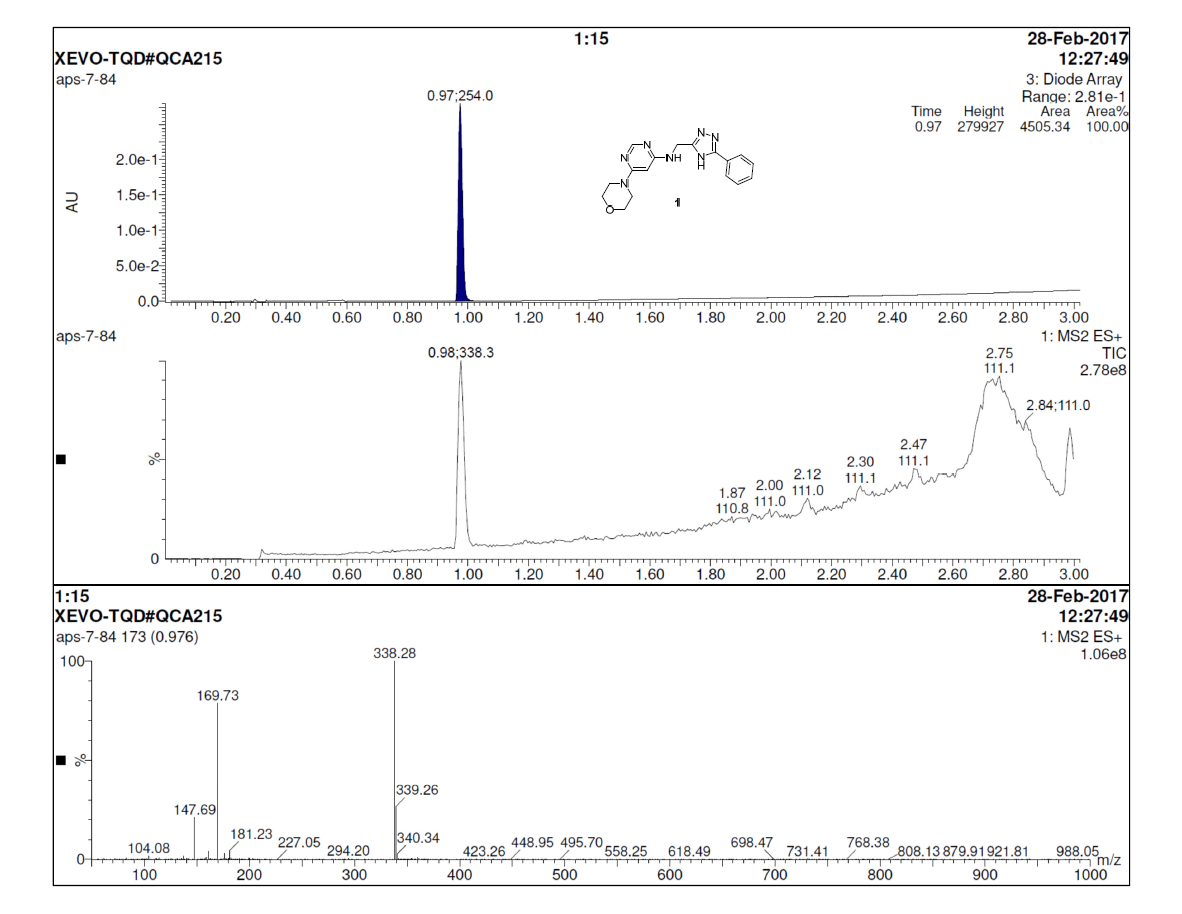


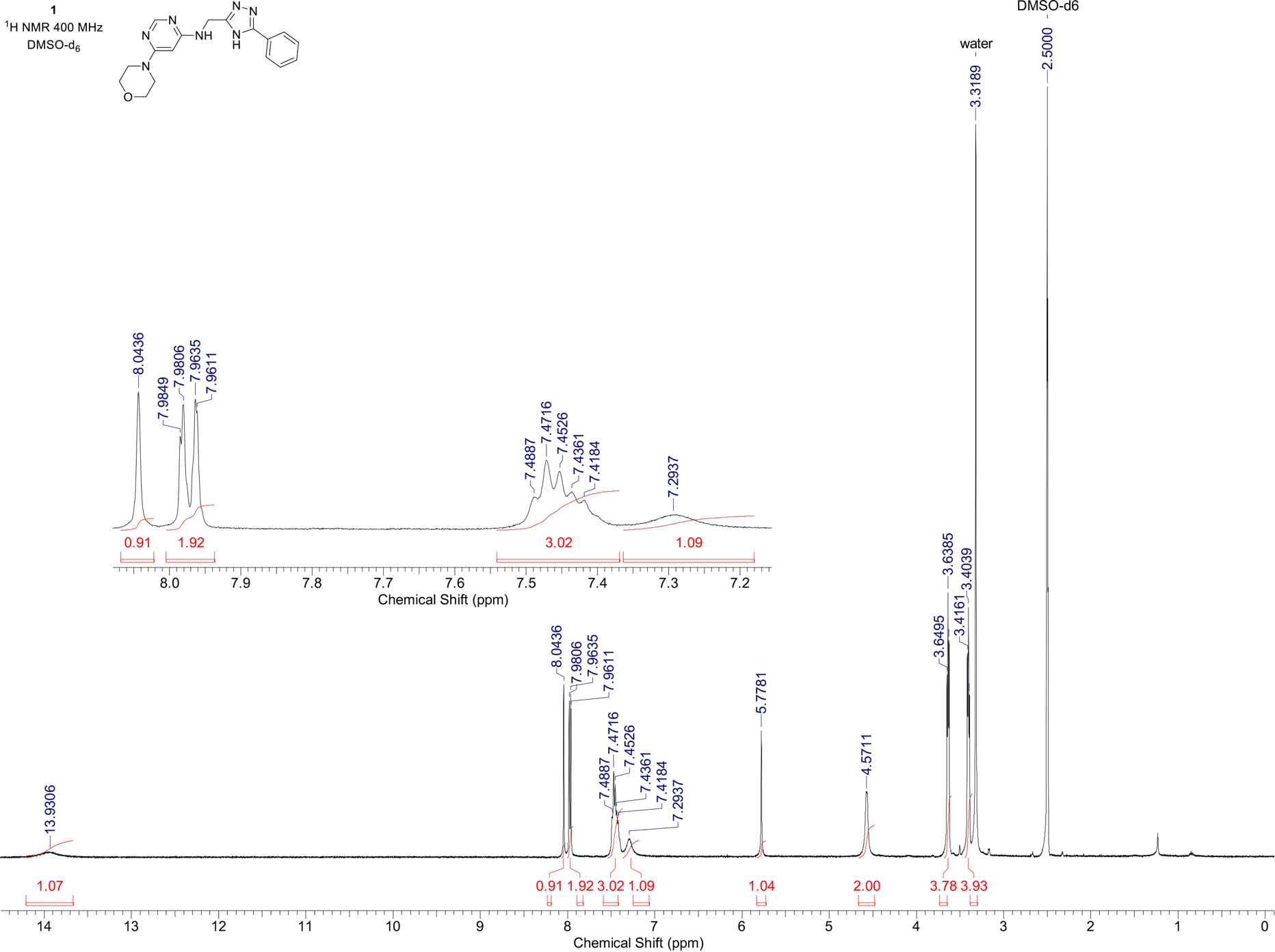

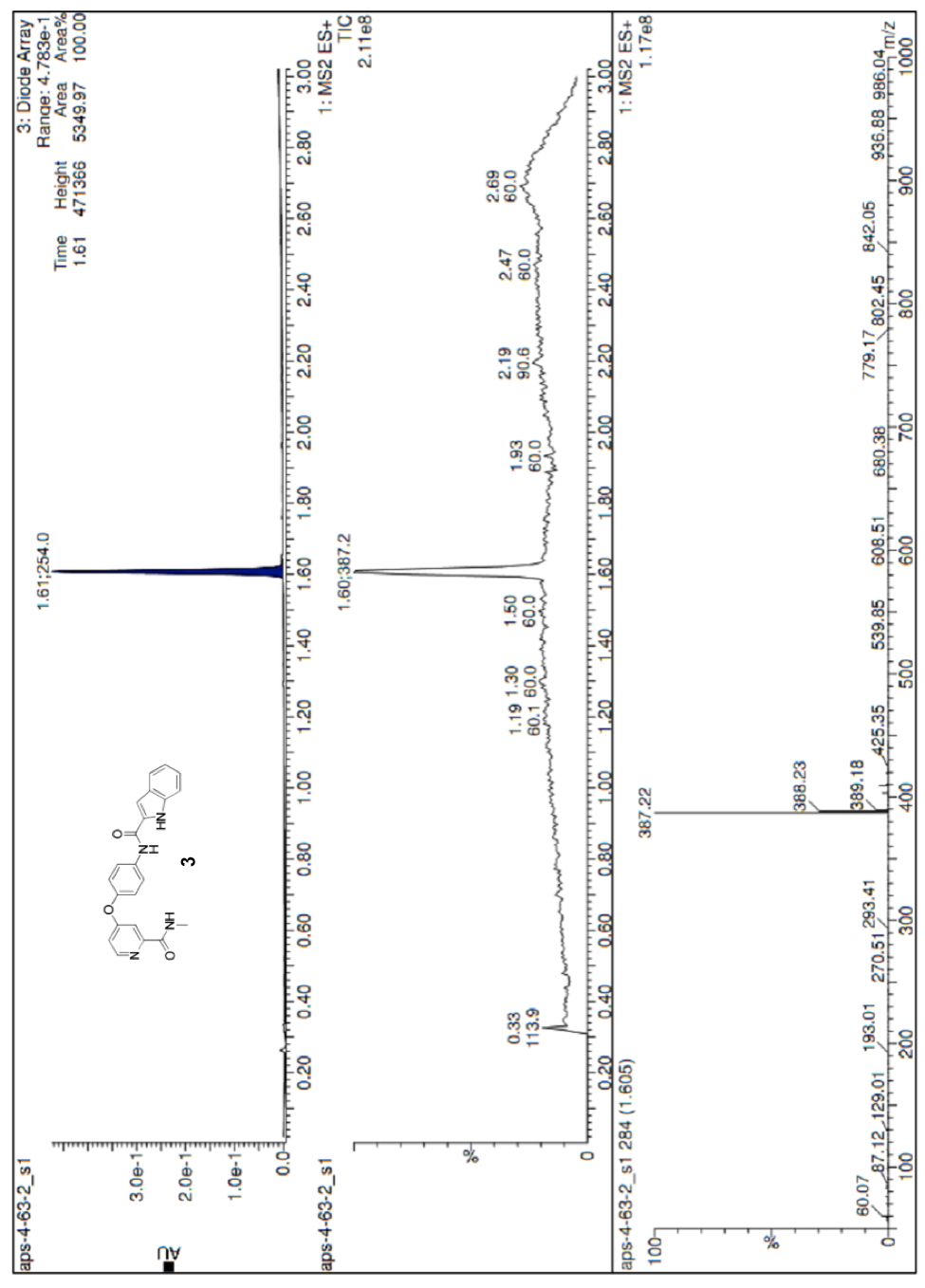

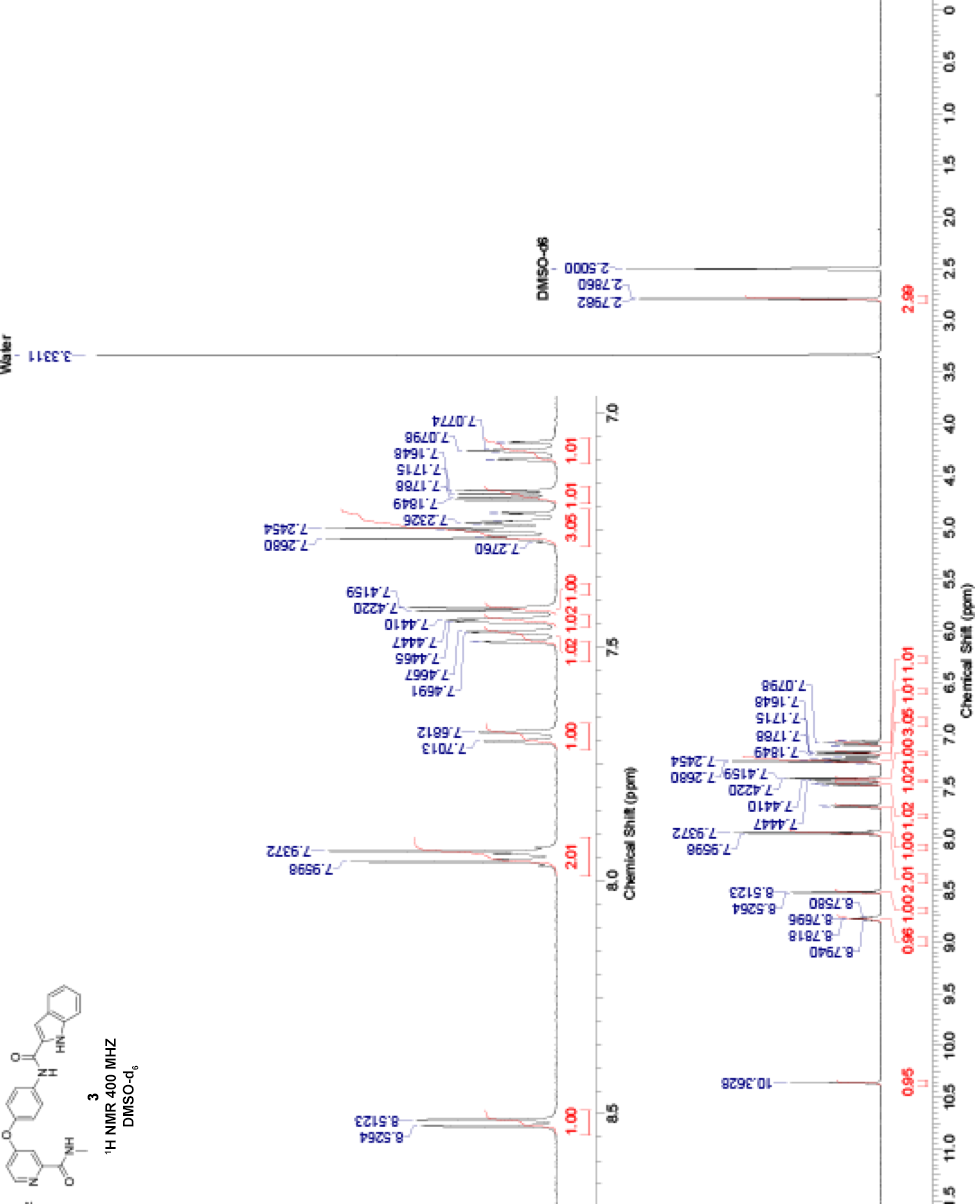

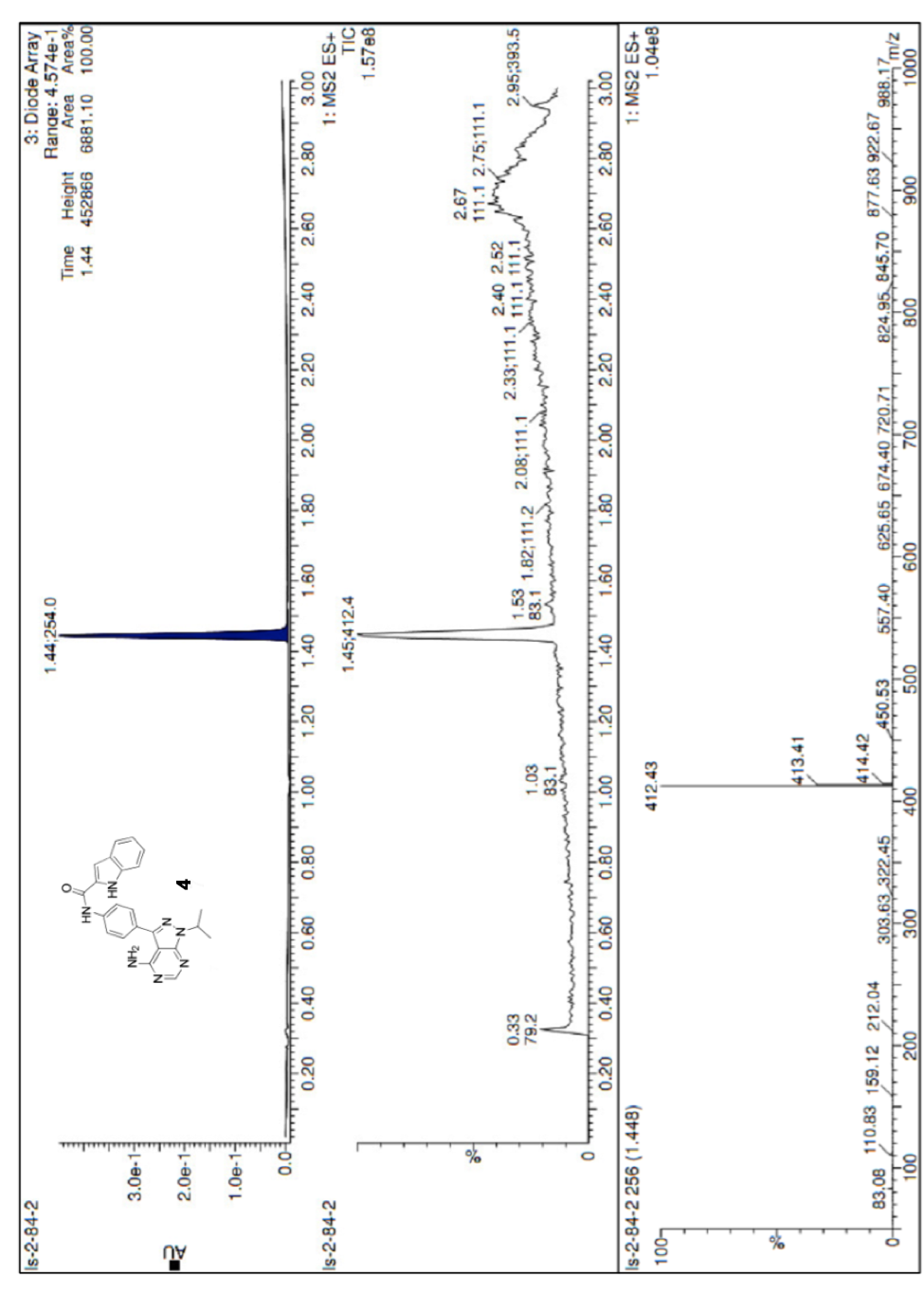


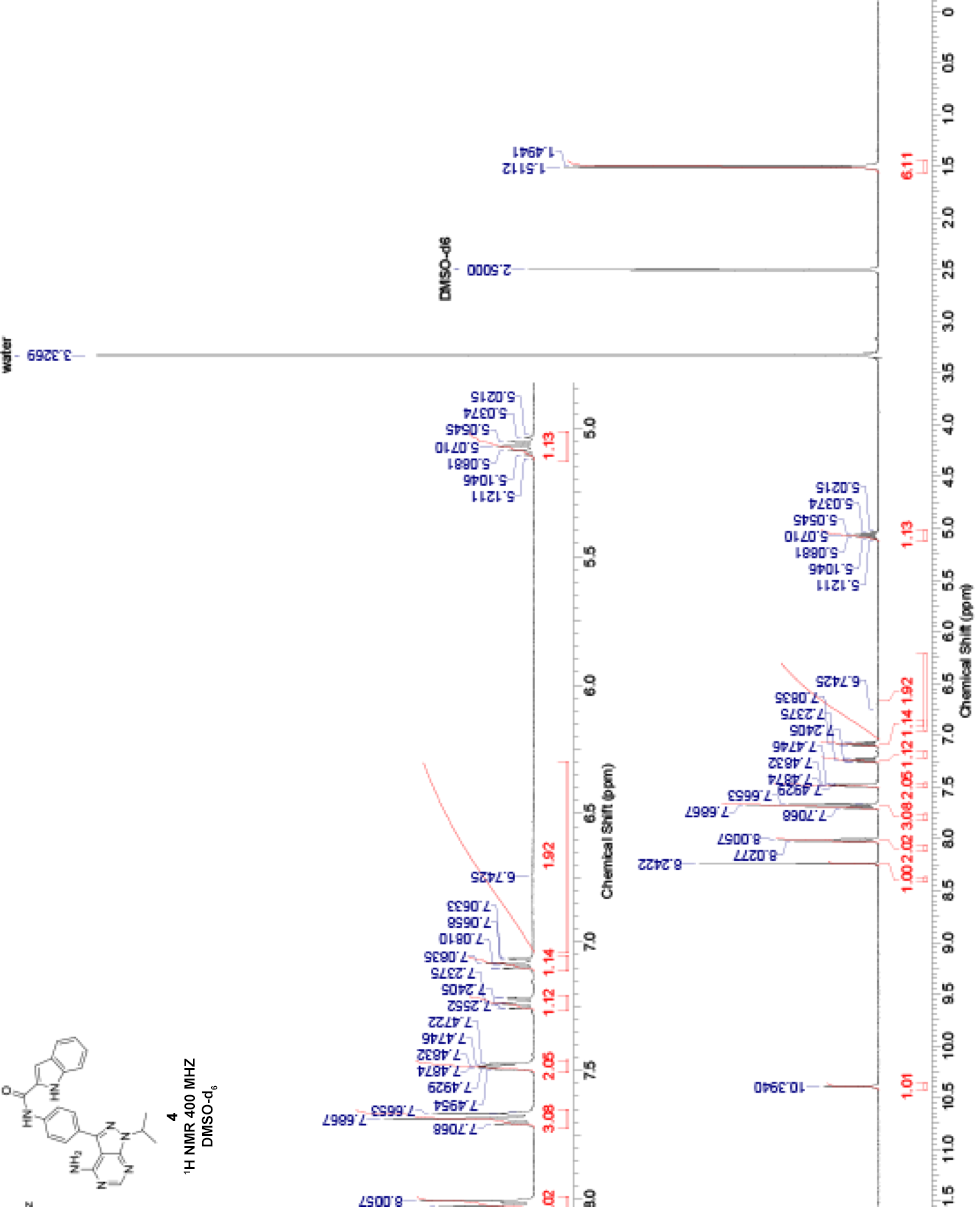


**REFERENCES**

1. Sonoshita M*, et al.* A whole-animal platform to advance a clinical kinase inhibitor into new disease space. *Nature chemical biology* **14**, 291-298 (2018).

2. Bankston DD, J.; Natero, R.; Riedl, B.; Monahan, M.-K.; Sibley, R. A scalable synthesis of BAY 43-9006: a potent Raf kinase inhibitor for the treatment of cancer. *Organic Process Research & Development* **6**, 5 (2002).

3. Dar AC, Das TK, Shokat KM, Cagan RL. Chemical genetic discovery of targets and anti-targets for cancer polypharmacology. *Nature* **486**, 80-84 (2012).
